# A Predictive Model for Yeast Cell Polarization in Pheromone Gradients

**DOI:** 10.1371/journal.pcbi.1004795

**Published:** 2016-04-14

**Authors:** Nicolas Muller, Matthieu Piel, Vincent Calvez, Raphaël Voituriez, Joana Gonçalves-Sá, Chin-Lin Guo, Xingyu Jiang, Andrew Murray, Nicolas Meunier

**Affiliations:** 1 MAP5, CNRS UMR 8145, Université Paris Descartes, Paris, France; 2 Institut Curie, CNRS UMR 144, Paris, France; 3 Unité de Mathématiques Pures et Appliquées, CNRS UMR 5669 and équipe-projet INRIA NUMED, École Normale Supérieure de Lyon, Lyon, France; 4 Laboratoire Jean Perrin and Laboratoire de Physique Théorique de la Matière Condensée, UMR 7600 CNRS /UPMC, Paris, France; 5 Molecular and Cell Biology and FAS Center for Systems Biology, Harvard University, Cambridge, Massachusetts, United States of America; 6 Institute of Physics, Academia Sinica, Taiwan; 7 Department of Chemistry and Chemical Biology, Harvard University, Cambridge, Massachusetts, United States of America; 8 CAS Key Laboratory for Biological Effects of Nanomaterials and Nanosafety, National Center for Nanoscience and Technology, Beijing, People’s Republic of China; University of Illinois at Urbana-Champaign, UNITED STATES

## Abstract

Budding yeast cells exist in two mating types, **a** and *α*, which use peptide pheromones to communicate with each other during mating. Mating depends on the ability of cells to polarize up pheromone gradients, but cells also respond to spatially uniform fields of pheromone by polarizing along a single axis. We used quantitative measurements of the response of **a** cells to *α*-factor to produce a predictive model of yeast polarization towards a pheromone gradient. We found that cells make a sharp transition between budding cycles and mating induced polarization and that they detect pheromone gradients accurately only over a narrow range of pheromone concentrations corresponding to this transition. We fit all the parameters of the mathematical model by using quantitative data on spontaneous polarization in uniform pheromone concentration. Once these parameters have been computed, and without any further fit, our model quantitatively predicts the yeast cell response to pheromone gradient providing an important step toward understanding how cells communicate with each other.

## Author Summary

Budding yeast cells exist in two mating types, **a** and *α*, which use peptide pheromones to communicate with each other during mating. Mating depends on the ability of cells to polarize up pheromone gradients, but cells also respond to spatially uniform fields of pheromone by polarizing along a single axis. We used quantitative measurements of the response of **a** cells to *α*-factor to produce a predictive model of yeast polarization towards a pheromone gradient. We found that cells make a sharp transition between budding cycles and mating induced polarization, and that they detect pheromone gradients accurately only over a narrow range of pheromone concentrations corresponding to this transition. We fit all the parameters of the mathematical model by using quantitative data on spontaneous polarization in uniform pheromone concentration. Once these parameters have been computed, and without any further fit, our model quantitatively predicts the yeast cell response to pheromone gradient providing an important step toward understanding how cells communicate with each other.

## Introduction

Many events in plant and animal development depend on the ability of cells to interact with only one of many potential partners. Examples include the interaction of neuronal growth cones with target cells [1], myotube fusion and vascular guidance [2, 3], the growth of pollen tubes to reach ovules [4], and the mating of many fungi, including budding yeast [5, 6]. These phenomena are based on the capacity of cells to polarize in response to spatially inhomogeneous external signals.

Cells polarize when they switch from behaving isotropically to showing a preferred axis [7]. While the biochemical basis of polarization can vary greatly, in its early stages polarization is always characterized by an inhomogeneous distribution of specific molecular markers. Cell polarization can be driven by internal or external asymmetries. As an example, an external gradient can cause chemotropism, the directed growth of cells along a chemical gradient. In mating yeast, the external signal is a pheromone gradient, which causes the cell to polarize its growth towards the source of pheromone produced by the mating partner, [6, 8–10].

**Fig 1:**
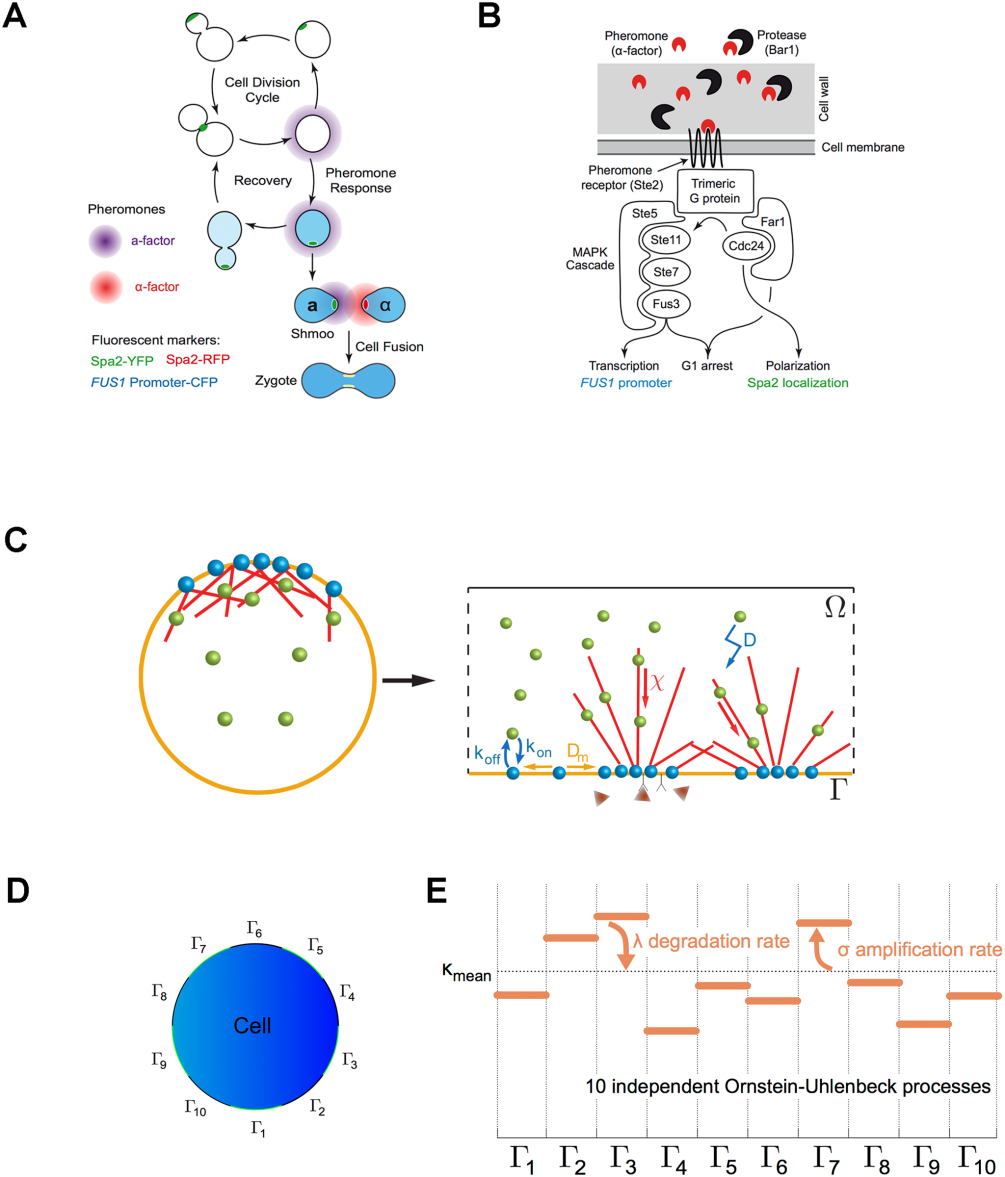
Basics of yeast mating and model. **A)** Mating as a developmental switch. Exposing haploid cells to pheromone makes them exit the cell division cycle, polarize towards, and fuse with a cell of the opposite mating type to form a diploid cell. Cells recover and resume budding if the pheromone signal disappears. The polarisome protein, Spa2 (shown in green and red), concentrates at the incipient bud site, at the bud tip as cells grow, at the bud neck during cytokinesis, and at the shmoo tip and fusion site during mating. We use the rate of accumulation of a fluorescent protein expressed from the FUS1 promoter, a gene induced by pheromone stimulation, as a readout of pheromone-induced signaling. **B)** The pheromone response pathway of **a** cells. *α*-factor diffuses through the cell wall and binds to the *α*-factor receptor (Ste2, a G protein-coupled receptor (GPCR)), which activates a trimeric G protein. The G protein recruits and activates two scaffolding proteins. One (Far1) recruits the actin polymerization machinery that leads to cell polarization and activates the kinase that activates the MAP kinase cascade. The other (Ste5) is responsible for the assembly of a MAP kinase cascade that activates the MAP kinase Fus3, leading to the induction of mating genes. Once phosphorylated by Fus3, Far1 arrests the cell cycle in G1. **C)** A two-dimensional model of cell polarization. The left panel shows a cell, the right a more detailed view introducing the various parameters in the model. Actin is polymerized into short filaments, that interact with each other and these are bundled together to form actin cables that cross the cell. The nucleation of filaments is proportional to both the local density of Cdc42 and to the concentration of pheromone. The endocytosis of markers at the membrane is described by very simple attachment/detachment dynamics (*k*_on_ and *k*_off_) and their diffusion in the plane of the membrane is described by a diffusion constant, *D*_*m*_, while their diffusion in the cytoplasm is described by a separate constant, *D*_*b*_. **D)** According to the fact that a polarisome occupies approximately 10% of the total membrane length, [12], we divide the membrane into 10 subregions (sectors). At each time point, *κ*(*t*,.), the parameter that describes the dynamics of the pheromone receptor, has a constant value within one segment. **E)** The temporal evolution of the effective pheromone receptor activity *κ* is ruled by an Ornstein-Uhlenbeck process. The parameter λ is the the noise damping. The parameter *σ* is the standard deviation of the instantaneous change of *κ*(*t*).

In nature, the budding yeast, *Saccharomyces cerevisiae*, is mostly found as diploid cells. Haploids exist in two mating types, **a** and *α*, which can proliferate asexually or mate with each other to form diploids. The **a** cells secrete **a**-factor and bear an *α*-factor receptor, whereas *α* cells secrete *α*-factor and detect **a**-factor. Both mating types express a common signal transduction pathway that is triggered by the binding of mating pheromones to their mating type-specific, G protein-coupled receptors. The signal passes through a MAP kinase cascade, inducing gene expression and polarized growth; polarization transforms ovoid cells into pear-shaped shmoos that grow towards each other (chemotropism) and fuse with each other at their tips (Fig 1A and 1B), [11]. The two pheromones are chemically distinct: *α*-factor is an unmodified 13 amino acid peptide, whereas **a**-factor is a 12 amino acid peptide that carries a C-terminal farnesyl group, which makes it extremely hydrophobic and difficult to work with. In wild type **a** cells, *α*-factor is degraded by a protease Bar1, which modifies the effective pheromone concentration experienced by the cell, but our experiments, which aim to expose cells to known pheromone concentrations are performed in **a** cells lacking Bar1.

The components of the signaling pathway and many of the connections between them are now well known: in the 1990s Hartwell and his colleagues showed that mating depends on communication between **a** and *α* cells (“courtship”) [12, 13], Segall [14] demonstrated that cells polarize up pheromone gradients and more recently the level of noise in the signaling pathway has been measured [15, 16]. Thanks to these studies, budding yeast has taken center stage for quantitative and modeling approaches to understanding of cell polarization. Importantly, yeast cells can also polarize in the absence of any gradient of signaling molecules. This happens once during each cell division cycle when a bud site is determined, and also when unbudded, haploid cells form a shmoo in response to a spatially uniform pheromone concentration. Shmooing and budding are distinct types of polarization. The choice of the budding site relies on historical marks as well as internal feedback loops; each division event leaves a localized scar with specific molecular markers (including the Bud1 protein) that localize the next polarization event. At very high pheromone concentrations, far above the K_d_ of the pheromone receptor, the same marks can be used to direct shmooing, because at such high concentration polarity markers accumulated at the budding site (historical mark) have no time to decay [12, 13]. But at physiological pheromone concentrations, cells shmoo randomly with respect to the direction they would have budded (Fig H in S1 Text). This independence from known historical marks makes shmooing in uniform fields a convenient phenomenon to develop and test models for polarization in response to external signals.

Several studies have proposed mathematical models that incorporate many aspects of the molecular mechanisms involved in pheromone-induced polarization. Although some of these models have been tested for their ability to fit quantitative data [17–24], they have not been quantitatively assessed for their ability to make accurate predictions with no additional free parameter. In this work we aimed at providing a simple and predictively useful mathematical description of how yeast cells respond to pheromone gradients. To test the predictive power of the model, we adopted the following strategy. We built a phenomenological model that used a minimal set of unknown parameters. This model was first introduced in [25], then studied in [26, 27] but not tested for its ability to predict experimental data. We used the polarization of cells exposed to an isotropic concentration of pheromone (no gradient) to fit the three unknown parameters of the model. Without further modifying these parameter values, we then used our model to predict the ability of yeast to polarize in the direction of pheromone gradients and compared the predictions to data for yeast polarizing in gradients formed in microfluidic devices. The simulated data matches fairly well with the experimental data, including the observation that, in pheromone gradient, cells can only polarize accurately over a narrow range of pheromone concentrations.

## Results

### Model description

We consider a two-dimensional model based on a long-range spatial coupling between sites on the membrane. Although the precise mechanism of such a coupling is still debated, transport of signaling molecules along actin filaments might be a good candidate to explain it. Although actin filaments are not essential to localize polarity markers in cell attempting to bud, they are necessary for stable pheromone-induced polarization [28, 29]. Our model is based on the active transport of components along cytoskeletal filaments, which directly or indirectly affect Cdc42 distribution. Cdc42, a positive regulator of actin filament nucleation, and actin filaments can generate a positive feedback loop in the following way: actin-based, active transport of molecules towards the membrane (mostly associated with vesicle transport) can modify the Cdc42 distribution which in turn determines the density of actin filaments at the membrane. There are numerous models for spontaneous polarization (see [9] for a discussion of the different possible feedback loops from a biological viewpoint). Polarization can be modeled by reaction-diffusion systems, Turing instabilities [30–33], recruitment of polarization molecules [34–39], and depletion of limiting components [40].

To minimize the number of parameters, we opt for a coarse-grained description of the actin cytoskeleton as an advection field accounting for long-range spatial coupling. We also consider a random motion of Cdc42 within the cytoplasm together with endocytosis at the membrane and we respectively denote by *n*(*t*, **x**) and *c*(*t*, **x**) the concentrations of Cdc42 and of actin filaments in the cytoplasm and by *μ*(*t*, *s*) the concentration of Cdc42 on the plasma membrane. The model reads as:

$$\begin{array}{c} \left\{\begin{array}{c} \partial_{t}n=D_{b}\;\Delta n-\chi \;\nabla \cdot \left(n\;\nabla c\right)\;,\;\text{for}∥x∥<R\;,\\ \partial_{t}\mu =D_{m}\;\partial_{ss}\mu +k_{\text{on}}\;n-k_{\text{off}}\;\mu \;,\;\text{for}∥x∥=R\;, \end{array}\right. \end{array}$$ (1)

where *k*_on_ and *k*_off_ are the attachment and detachment rates of Cdc42 at the membrane and the cell is described by a disk of radius *R*. Active transport is modeled as the gradient of the concentration of filaments in the cytoplasm, *χ*∇*c*. The parameter *χ* can be interpreted as a correlation length and the term *χ*∇*c* as a long-range spatial coupling. We suppose that the nucleation of new filaments occurs at the plasma membrane, under the combined action of Cdc42 and the pheromone signal (Fig 1C). After a dimensional analysis, the model that describes the cytoskeletal density reads as:

$$\begin{array}{c} \left\{\begin{array}{c} -\Delta \;c+\eta \;c=0\;,\;\text{for}∥x∥<R\;,\\ -\nabla c\;\cdot \;{\vec{e}}_{x}=\kappa \;\frac{S}{S_{0}+S}\;\mu \;,\;\text{for}∥x∥=R\;, \end{array}\right. \end{array}$$ (2)

where ${\vec{e}}_{x}$ is the unit outward normal vector, *S* is the pheromone-generated signal at the membrane, and *c* and *κ* are dimensionless numbers. The Michaelis-Menten ratio *S*/(*S*_0_ + *S*) accounts for the maximal number of receptors on the cell membrane, see [41]. In this models (1) and (2), *μ* is the concentration of the total Cdc42 on the membrane, including non-active Cdc42, and it depends on actin cables, described by *c*. Then *S* gives the activation of the membrane bound Cdc42 and leads to actin polymerization (and thus controls *c*). A more realistic model might have been to explicitly distinguish active and inactive Cdc42 with two different variables, which would have introduce more parameters. In our model total membrane Cdc42 is *μ* and active Cdc42 is *Sμ*. Our approach has the advantage of minimizing the number of parameters while accounting for Cdc42 activation.

These equations are complemented by initial conditions and by an additional boundary condition on the cell membrane which guarantees the conservation of the total Cdc42 pool (S1 Text).

First, we tried to fix the parameter values from literature and dimensioning arguments. The sensitivity of the model output to the choice of these parameters will be described and discussed later on in the article. We observed that changing the concentration of actin filaments in the cytoplasm *c* into *c*/*κ* in Eq (2) leads to replace the advection speed *χ* by *κχ* in Eq (1). Hence, in the absence of noise, *κ* can be fixed arbitrarily, and its value will be discussed below. The observation that, without any pheromone, 13% of the Cdc42 molecules are on the membrane at steady state [36, 42], sets the value of the ratio of attachment and detachment rates of Cdc42 at the membrane, *k*_on_/*k*_off_ ~ 0.16*μ*m (S1 Text). This simple model has only two unknown parameters *k*_off_, the endocytosis rate, and *χ*, the advection speed accounting for long-range spatial correlation (Table 1 and S1 Text). However this model accounts for a number of molecular processes in an effective manner, which we describe now. At the molecular scale, processes that take place once the pheromone binds some receptor molecule on the cell membrane mostly rely on chemical reactions involving a small number of molecules, such as the trimeric G proteins that interact with the pheromone receptor and regulate Cdc42. The activation and inactivation of the pheromone receptors, which reflects a mixture of binding, unbinding, lateral diffusion, and endocytosis are summarized by the parameter *κ*. Importantly, the activation and inactivation might fluctuate over time and space, [24, 43–45]. These fluctuations cannot be accounted for by a constant parameter, hence *κ* takes non constant values. More precisely, we assume that the phenomenological parameter *κ* which we call the effective pheromone receptor activity is described by a stochastic process. We opt for the minimal Ornstein-Uhlenbeck stochastic process. The stationary measure is indeed a Gaussian distribution with only two parameters which will be determined from experiments (S1 Text). The spatial correlation length of the fluctuations of this stochastic process (Fig 1D and 1E), can be fixed considering that a polarisome (the local concentration of proteins that induces cell polarization) occupies approximately 10% of the total membrane equatorial permiter length, [42] (S1 Text). The damping coefficient of the noise, λ (Fig 1E), which reflects the combined action of pheromone binding and unbinding, and the lateral diffusion and endocytosis of the receptor, is dominated by the fastest of these processes. Physical arguments provide bounds for 1/λ, which corresponds to a relaxation delay: the literature, see [46] e.g., argues that the half time for receptor endocytosis, at least at high pheromone concentrations, is on the order of ten to twenty minutes. Here we suppose that 1/λ ∼ 10 min. In addition to the previous source in noise in the effective pheromone receptor activity, *κ*, whose variation represents noise in both space and time within a single cell, we consider a second source of noise to describe the cell-to-cell variability in the total Cdc42 pool *M*. Indeed the value of the total Cdc42 pool might fluctuate between cells. We observe that changing the three parameters (*M*, *κ*, *χ*), which are respectively the total Cdc42 pool, the effective pheromone receptor activity and the advection speed, in (*αM*, *κ*/*α*, *χ*) does not affect the solution (*n*, *μ*) of the models (1)–(2) which describes the Cdc42 concentration. Consequently we consider that the mean effective pheromone receptor activity, *κ*_mean_, follows a normal distribution with mean arbitrarily fixed to the value 13% × *M* and standard deviation *δ*. This latter parameter is unknown and describes a longer term variation between cells.

**Table 1:** The sixteen parameters involved in the model with their definitions, their values, and the source from which the value is derived.

*R*	Cell radius	2.5 *μ*m	[66]
*R*_*n*_	Nuclear radius	1 *μ*m	[66]
*D*_*b*_	Bulk (cytoplasmic) diffusion coefficient	1.4 *μ*m^2/^s	[32]
*M*	Total Cdc42 content	10^3^	[42]
*S*_0_	*K*_*d*_ of pheromone receptor	6 nM	[41]
*η*	Actin depolymerisation rate	10^−3^ s^−1^	[67]
*k*_off_	Endocytosis rate	0.12 s^−1^	Fitted
*k*_on_	Exocytosis rate	*k*_on_ = *k*_off_ × (0.16 *μ*m)	[36, 42]
*D*_*m*_	Membrane diffusion coefficient	$\sqrt{D_{m}/k_{\text{off}}}∼0.37\;\mu$m	[42]
*κ*_mean_	Mean effective pheromone receptor activity	13, it is arbitrary since only *κχ* affects the system	Free
2*πR*/*N*	Correlation length of the noise for *κ*	10% of the cell perimeter	[42]
λ	Damping coefficient of *κ*	λ^−1^∼ 10 mn	[68]
*σ*	Standard deviation of *κ*	*σ*^2^/2λ = 13, stationary noise intensity assumption	[69]
*δ*	Cell-to-cell variability	0.2	Fitted
*χ*	Actin-based transport coefficient	2.5 ×10^5^ *μ*m^2^/s	Fitted
(*n*_0_(**x**), *μ*_0_(*s*))	Initial condition	Steady state at *S* = 0 nM	Nature of the experiments and [36, 42]

Finally, the complete model including the noise on the effective pheromone receptor activity involves three unknown parameters, two of them have a significant impact, the advection speed accounting for long-range spatial correlation, *χ*, and the endocytosis rate, *k*_off_ (Table 1 and S1 Text).

We now present the experiments that have been designed to estimate the values of the three missing parameters: the advection speed accounting for long-range spatial correlation, *χ*, the Cdc42 endocytosis rate, *k*_off_, and the cell-to-cell variability, *δ*.

### Cells respond precisely to pheromone

**Fig 2:**
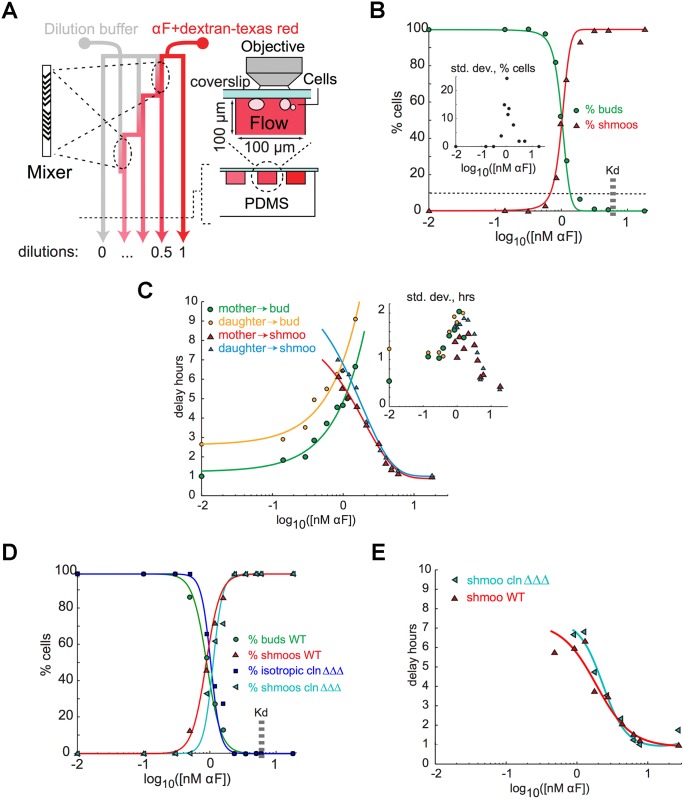
The response of bar1Δ cells response to homogenous stimulation by *α*-factor. **A)** Schematic of a device to produce a range of pheromone concentrations by using chaotic mixers in dilution chambers. The diagram shows a plan view (left) and cross sections at two magnifications (right). Structured micro channels allow fast mixing and thus permit serial dilutions in a small device. A lectin (concanavalin A) binds yeast cells to the coverslip that forms the roof of the chamber. They receive a constant flow of pheromone, allowing them to be exposed to concentrations that are stable over several hours and can be measured by quantifying the emission of a fluorescent dextran mixed with the pheromone (see Experimental Procedures in S1 Text and Fig A in S1 Text for more details). **B)** The behavior of cells in micro-channels at various pheromone concentrations. We assessed cell behavior at each pheromone concentration by following cells over time and overlaying differential interference contrast and Spa2-YFP images (see Fig B in S1 Text for images). The graph quantifies the bud/shmoo transition in spatially uniform fields of pheromone and summarizes data from about 4000 cells (on each curve (red and green), at least 160 cells (MP 384) were used to obtain the averages shown for each pheromone dose) in seven independent dilution chambers. The inset shows the standard deviation of the fraction of different events between experiments. The measured dissociation constant of *α*-factor from Ste2 is indicated (*K*_*d*_) [41]. The half maximal point of the sigmoidal fit is 1.02 ± 0.03 nM and the Hill coefficient for the transition between budding and shmooing is 6.5 ± 0.6 (95% confidence interval). **C)** Quantification of polarization delays. Time was measured from the end of the first cytokinesis after the onset of pheromone treatment. Cells were considered polarized when a stable focused Spa2 cap was formed. Only those cells whose progenitors had completed cytokinesis during the first three hours after the start of pheromone treatment were considered. At least 52 cells (MP 384) were used to obtain the averages shown for each point. **D and E)** Cells arrested in G1 by G1 cyclin depletion were placed in exponential dilution chambers together with wild type cycling cells (using a fluorescent cell wall marker to distinguish the two strains). Experiments and measures are similar to those shown in B) and C). The transition to shmooing occurs at exactly the same concentration for G1 arrested and cycling cells (G1 arrested cells that do not shmoo grow isotropically, forming large spherical cells). Because Cln-depleted cannot leave G1 and cells arrested in G1 never polarize except when they form a shmoo, we do not show the delay before rebudding for wild type cells. The delay before shmooing is similar for cycling cells and for G1-arrested cells. In graph D) each point corresponds to an average value computed over 66 cells (MP 384 and MP 1333) and in graph E) over 52 cells (MP 384 and MP 1333).

Budding yeast can polarize spontaneously in the absence of an external pheromone gradient, [9, 34–36, 42, 47]. To estimate the values of the missing parameters involved in our model we used data from measuring the fraction of cells which polarize and the delay before polarization as a function of the applied, spatially uniform pheromone concentration. Previous work has shown that transition between budding and shmooing occurs at a low pheromone concentration, around 1 nM [44, 48], and it is known that the delay before cells shmoo rises as the pheromone concentration falls. To precisely measure the fraction of cells that polarized and the delay before polarization, we used a microfluidic device to apply stable low pheromone concentrations for long periods (up to 10 hours) and followed individual cells by light microscopy (Fig 2A, S1 Text and Fig A in S1 Text).

We followed cell polarization by fluorescently tagging Spa2, a component of the polarisome [49–51]. The polarisome is located at sites of polarized cell growth (the tips of buds and shmoos, and the site of cell division [52]), and appears there before cells switch from isotropic to anisotropic growth.

We observed that cells shmooed at concentrations above 1nM pheromone (Fig 2B and Fig B in S1 Text). Like others [48, 53, 54], we saw little cell-to-cell variability: at 0.6 nM *α*-factor, 96% of cells budded after a delay; at 1 nM, buds and shmoos were equally common; and at 2 nM, 93% of the cells shmooed (Fig 2B and Fig B in S1 Text). The delay before cells budded rose as the pheromone concentration rose, but the delay before they shmooed fell (Fig 2C). Once cells had shmooed, they did not subsequently bud, even after 16 hours of incubation with pheromone (see Fig B in S1 Text and MP unpublished data). The failure of cells to shmoo at low pheromone concentrations (< 1 nM) could reflect competition between budding and shmooing. We eliminated this possibility by applying a pheromone-independent G1 arrest and then exposing cells to *α*-factor. Cells that had been arrested by removing their G1 cyclins required the same concentration of *α*-factor to induce shmooing as cycling cells (Fig 2D) and the timing for polarization was also similar (Fig 2E).

### Parameter estimation from uniform pheromone concentration experiments

In the experiments described above, cells were suddenly exposed, at time zero, to a wide range of temporally stable concentrations of pheromone. Therefore, in our numerical simulations we assumed an initial distribution of Cdc42 corresponding to a steady state pheromone concentration, *S* = 0 nM, at the cell membrane (S1 Text). Furthermore, we considered that 13% of the Cdc42 molecules were on the membrane, see [36, 42].

**Fig 3:**
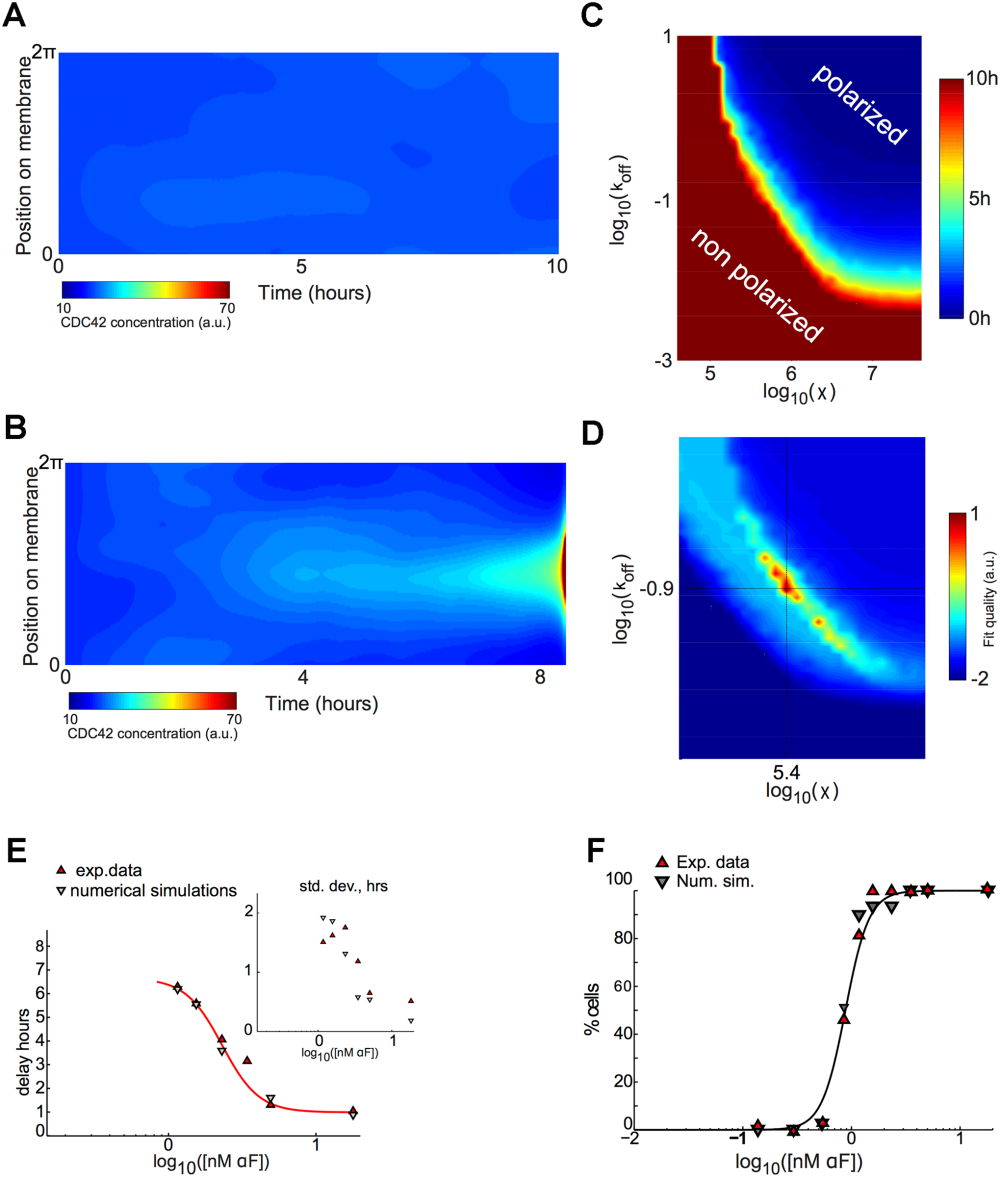
Model behavior and model parameters value selection. **A and B)** Increasing the strength of pheromone stimulation (S) leads to increasing levels of spatial segregation and for a given *S*, depending on the spatial correlation length *χ* and the endocytosis rate *k*_off_ the cell will polarize or not. Shown are kymographs from simulations (*y* axis, membrane position; *x* axis, time). Particle density is in absolute value. On the left we see an unpolarized cell and a polarized one on the right. **C)** Representation of mean time polarization isovalues for *S* = 1 nM as a function of the spatial correlation length *χ* and the endocytosis rate *k*_off_. If the numerical simulations did not lead to a polarized state before 10 hours, we considered the cell to be non-polarized. **D)** Representation of the cost function (labeled as fit quality) depending on *χ* and *k*_off_, allowing us to determine the optimal pair of values (*χ*, *k*_off_ that fit the data (Fig 2C). **E)** For the optimal values of *χ*^opt^ = 2.5 × 10^5^ *μ*m^2^/s, $k_{\text{off}}^{\text{opt}}=0.12$ s^−1^ and *δ*^opt^ = 0.2, we show the simulated timing of polarization for solutions to our model under varying pheromone concentration compared to the experimental data for polarization timing. The inset shows the standard deviation of the timing of polarization for solutions to our model under varying pheromone concentration compared to the experimental data for polarization timing. **F)** For the same optimal parameters $\left(\chi^{\text{opt}},k_{\text{off}}^{\text{opt}},\delta^{\text{opt}}\right)$, we represent the numerical fraction of cells which polarize under varying pheromone concentration. Before fitting the model parameters with the data, we defined the numerical criterion for polarization. Following [42], we considered that polarization occurred when more than 50% of the total membrane protein pool was located in a window of 10% of the membrane. Hence, a numerical simulation corresponded to a polarization state if there existed a time for which more than 50% of the total membrane Cdc42 was located in less than 10% of the perimeter of the cell boundary (S1 Text).

For a concentration of pheromone *S* = 1 nM, by using standard finite volume method (for more details see [55] and S1 Text), we ran simulations with the model for several values of the parameter accounting for long-range spatial correlation, *χ*, and the Cdc42 endocytosis rate, *k*_off_. In each numerical simulation we computed the cytoplasmic and membrane Cdc42 concentrations. We observed that different parameter values gave different regimes: homogeneous (unpolarized state) and heterogeneous (polarized state) membrane Cdc42 concentrations, (Fig 3A and 3B).

Next, for different values of the advection speed *χ* accounting for long-range spatial correlation and of the Cdc42 endocytosis rate *k*_off_ and for each concentration of pheromone (on a logarithmic scale, from log *S* = 0 to 1 with an increment of 0.25, where *S* is expressed in nM), we ran 300 simulations with random values of the effective pheromone receptor activity *κ*, with different values of the longer term variation between cells *δ*, and we computed the time required for polarization (Fig 3C). By using an optimization procedure (Fig 3D and S1 Text), we found that the best values of the spatial correlation length, the Cdc42 endocytosis rate and the cell-to-cell variability were respectively *χ*^opt^ = 2.5 × 10^5^ *μ*m^2^/s, $k_{\text{off}}^{\text{opt}}=0.12$ s^−1^ and *δ*^opt^ = 0.2. For these three optimized parameter values we have plotted the average numerical polarization delay values (Fig 3E) and the ratio of cells that polarized in a time span of 10 hours (Fig 3F). The good agreement between the simulations and the experimental data, for both the mean and the standard deviation, demonstrated that we had built a model that quantitatively described yeast cell behavior in uniform pheromone concentrations.

With these experiments we were able to determine the values of all of the three unknown parameters. We next asked whether our model, without changing any parameter value, could quantitavely predict cells’ response to pheromone gradients.

### Directional response to pheromone gradient

**Fig 4:**
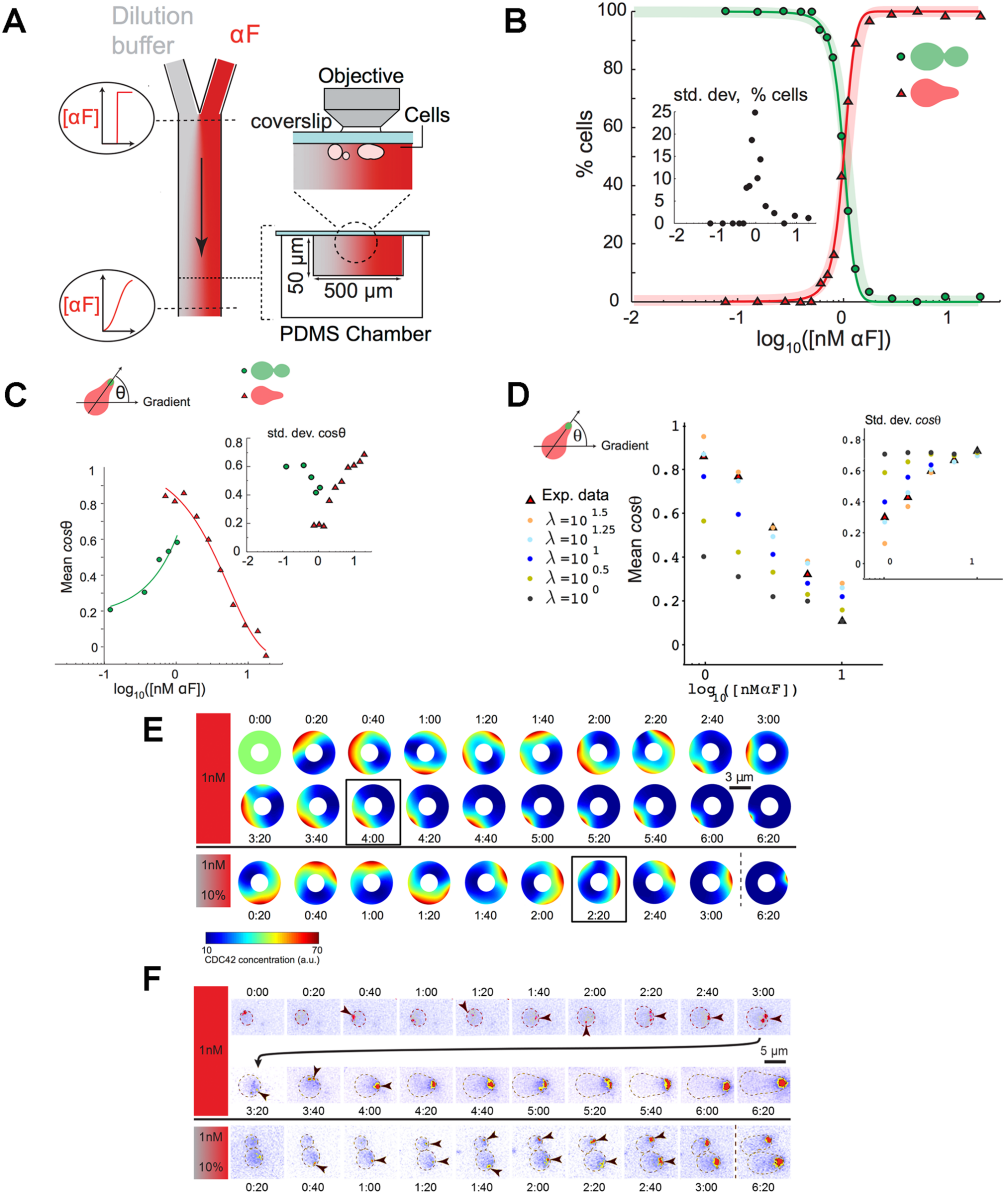
Response to pheromone gradients and model prediction. **A)** Producing pheromone gradients in a laminar flow chamber. Pheromone mixed with a fluorescent dextran and a dilution buffer enters through two ports and diffusion between the two fluid streams creates a temporally stable, gradient (see Experimental Procedures in S1 Text, and Fig C in S1 Text for more details). The left hand view is from the top of the apparatus and right hand views are two different magnifications of a cross-section, showing the cells attached to the coverslip that forms the roof of the chamber. **B)** The transition between budding and shmooing, quantified as in Fig 2B. The lightly shaded, thick curves show the data from spatially uniform pheromone concentrations (Fig 2B) for comparison. For every cell, the difference in concentration between the two edges of the cell was > 5% (expressed relative to the mean concentration that the cell experienced). The inset shows the standard deviation. **C)** The accuracy of gradient detection as a function of pheromone concentration. Accuracy is defined as the mean cosine of the angle between the gradient and the line that connects the Spa2 polar cap to the center of the cell. **D)** The accuracy of gradient detection as a function of pheromone concentration for solutions to our model. For different damping coefficients λ, the gradient detection and the standard deviation were computed. We observe that the value of λ which fits the data the best is 10^1.25^ which means 1/λ_opt_ ≈ 3 mn. **E)** We show the timing of polarization in representative numerical simulations of cells exposed to a uniform field and gradient field of *α*-factor (images are shown every 20 minutes). **F)** Comparing the timing of polarization in individual cells exposed to a uniform field (top) and a gradient (bottom) of *α*-factor. Images were taken every 20 minutes and pseudocolored to indicate the intensity of Spa2-YFP fluorescence. Note the small unstable Spa2 spots (arrowheads) that appeared in the homogenously stimulated cell long before a stable polar cap, which took four hours to develop. In contrast, in the gradient, a small Spa2 spot first appeared in the direction of the gradient and then gradually grew stronger allowing the cell polarize much faster.

We used laminar flow chambers to measure the response of *bar1*Δ cells to well-defined pheromone gradients (Fig 4A and Figs C, D and E in S1 Text). These experiments led to five conclusions: i) The transition between budding and shmooing occurred at the same concentration (1 nM) as it does for cells in homogeneous pheromone concentrations (compare Fig 4B with Fig 2B), ii) cells could only detect gradients accurately over a narrow range, with mean pheromone concentrations ranging from 0.7 to 2.5 nM, (Fig 4C), iii) the accuracy of gradient detection fell as the gradient became shallower than a 5% difference in concentration between the two sides of the cell (Fig E in S1 Text), iv) even extremely steep gradients could not increase the range of concentrations that allowed *bar1*Δ cells to respond accurately to gradients (Fig E in S1 Text), and v) cells detected gradients most precisely at the mean concentration (1 nM) that equalled the lowest concentration that induced shmooing in an isotropic field of pheromone (compare Fig 2B with Fig 4C).

We simulated mean pheromone concentrations varying, on a logarithmic scale, from log *S* = 0 to 1 with an increment of 0.25, where *S* is expressed in nM, with a concentration gradient across the cell equal to 10% of the mean pheromone concentration (the slope for which we had the most accurate experimental data). For each pheromone level we ran 300 simulations for our model with the parameter values obtained previously and with the same constant initial condition. Note that the effective pheromone receptor activity *κ* takes random values since it is modeled by a stochastic process as was previously explained. We computed the angle between the localization of the polar cap and the direction of the pheromone gradient at the first time point corresponding to a polarized state (according to our numerical criterion). The results were then averaged for a given pheromone concentration (Fig H in S1 Text). These simulations recapitulated the main experimental observations. In particular, polarization occurred at 1 nM, and was accurately oriented over a narrow range of mean concentration with an optimal detection of the gradient occurring at the mean concentration (1 nM) that equaled the lowest concentration that induced numerical polarization in an isotropic field of pheromone. This demonstrates that our simple phenomenological model is rich enough to quantitatively predict the response of yeast cells to pheromone gradients.

### Sensitivity analysis

To assess the robustness of the model output to the parameter values, we performed a sensitivity analysis for the unknown parameters involved in the model, see Table 1 for a description of all the parameters involved in the model. We fixed the parameters whose values were available in the literature: the cell radius *R*, the cell nucleus radius *R*_*n*_, the bulk diffusion coefficient of Cdc42 *D*_*b*_, the diffusion coefficient of Cdc42 on the plasma membrane *D*_*m*_, the actin depolymerisation rate *η* and the effective *k*_d_ of the pheromone receptor *S*_0_. Moreover, we observed that varying the parameters *η* and *R*_n_ had a negligible effect. Furthermore, since changing the value of the total Cdc42 pool *M* was equivalent to changing the value of either the effective parameter *κ*_mean_, which reflects the dynamics of the signal transduction cascade, or the coefficient which can be interpreted as a spatial correlation length *χ*, we considered the value of *M* as fixed. We then assessed the sensitivity of the model predictions to the endocytosis and exocytosis rates, to *χ*, to the initial conditions, and to the parameters associated to the stochastic process describing the fluctuations of the effective pheromone receptor activity, namely the spatial correlation length 2*π*/*N*, the damping coefficient λ, the standard deviation *σ*, of the noise intensity and the standard deviation of the fluctuations of the mean effective pheromone receptor activity *κ*_mean_ which is also the cell-to-cell variability *δ*.

The model appeared to be very sensitive to the two unknown parameters *χ*, *k*_off_ describing the long-range spatial correlation activity and the endocytosis rate (and hence the exocytosis rate *k*_on_ which is derived from these two). Indeed, the experimental values for the polarization delay and the fraction of cells that polarized are matched only for a very narrow range of values of these two parameters (Fig 3A–3D). When the parameters were not in this narrow range, the model displayed either no polarization at all or the wrong polarization dynamics in response to uniform pheromone concentration and hence did not have any predictive power for the response to gradients.

The parameter *δ* which models cell-to-cell variability was necessary to fit the standard deviation (Fig 2B) and it had some quantitative effect on the sharpness of the fraction of cells which polarize in uniform pheromone concentration (compare Fig 3F and Fig F in S1 Text).

The two parameters λ and *σ*, which are respectively the damping coefficient and the standard deviation, involved in the Ornstein-Uhlenbeck process describing the effective pheromone receptor activity *κ*, and which are linked by the relation *σ*^2^/2λ = *κ*_mean_, had no effect in the case of uniform pheromone concentration (Fig F in S1 Text). But they had some quantitative, but not qualitative impact when considering variations in a realistic range of values, 1/λ varying between 2 minutes and one hour (Fig 4D), and qualitative impact when considering very large variations in an unrealistic range of values (Fig H in S1 Text), on the model prediction in the case of the response to pheromone gradients. Both the quality of the gradient detection and the drop in the precision of orientation with increasing pheromone concentration were affected. We observed that performing numerical simulations with different parameter choices for the half life 1/λ from the range 1 to 20 mn has a qualitative but not quantitative effect on the predictions of the model and that the optimal value was 3 mn (Fig 4D and Fig H in S1 Text).

The last parameter involved in the stochastic process *κ* is the correlation length of the noise. Numerically it is modeled by the number of sectors *N* which was assumed so far to be 10. Changing this parameter had no effect on the polarization delay (nor on the fraction of cells which polarize) in uniform pheromone concentration (Fig F in S1 Text) while it had a similar effect as the damping coefficient λ on gradient response.

Finally we observed that the initial conditions also had a greater impact on gradient detection than on the polarization delay in uniform concentration (Fig G in S1 Text). When the initial conditions did not correspond to a homogeneous steady state, the predicted gradient detection did not depend on the pheromone concentration (the gradient was either always or never detected).

In conclusion, we observed that only a small number of parameters significantly affected the model output. The model was particularly sensitive to the values of the two parameters, *χ*, the spatial correlation length and *k*_off_, the endocytosis rate, that were estimated from the experiments on cell response to uniform pheromone concentration. The model was also sensitive to the values of the parameters involved in the stochastic process *κ*, namely λ, *σ* and *N*. The values of these latter parameters were given by physical arguments.

## Discussion

Diffusion, long-range spatial coupling, and receptor endocytosis have all been shown to contribute to the establishment and dynamic maintenance of polarized membrane proteins during shmoo formation. Following earlier work [25–27], we considered a mathematical model to describe and study these three mechanisms for membrane-protein redistribution during polarity establishment in response to mating pheromone. This model, which relies on limiting amount of critical components and describes the flux of Cdc42, incorporates stochasticity in order to describe the large fluctuations that might appear when pheromone molecules bind a small number of receptors on the cell membrane. All but three parameters of the model were computed using the literature or physical considerations. We then fitted the remaining parameters using quantitative measurements of cell response to uniform pheromone concentration. Using this complete set of parameters, our model quantitatively predicted directed cell polarization in pheromone gradients.

Indeed cell spontaneous polarization and cell response to gradients are very different biological experiments. For experiments in gradient, there are three outputs instead of two in uniform concentration experiments: polarization threshold, polarization timing and direction of polarization. Our experiments showed that there is a correlation between the polarization threshold and the direction of polarization: cells polarize accurately towards the gradient only at pheromone concentration near the threshold of polarization. Like previous studies, [22] e.g., we found that yeast detect gradients better at low average pheromone concentrations. But in our case, in the absence of Bar1 proteins, gradient sensing did not occur over a large range of pheromone values. Furthermore, our model, which was fitted only based on information coming from response to homogeneous pheromone concentration, was able to predict this unexpected experimental observation.

The most striking result of our study is the predictive power of a simple phenomenological model. In the set of experiments used to compute the values of the unknown parameters, only two quantities were considered, the fraction of polarized cells and the timing of polarization. These experiments, performed in homogeneous pheromone concentrations, did not contain any information about the capacity of cells to detect gradients, which relies on the comparison of concentrations at different points at the cell surface. In most other studies, the models are validated based on qualitative agreement or by adjusting free parameters to reproduce quantitative experimental data. In this study we go one step further in the validation of our model by quantitatively predicting the output of a different experiment than the one used to adjust the free parameters. This gives us confidence that our model can be used as a prototypical phenomenological framework for cell polarization in response to external signals.

The simplest interpretation for the origin of the model’s predictive power is that the experimental data obtained with the homogeneous pheromone concentrations gives the probability and the timing of polarization for each pheromone concentration. This data locally fixes the behavior of each segment of the cell periphery depending on the pheromone concentration it experiences in the gradient, leading to the threshold behavior observed (cells only polarize accurately if at least part of their periphery is below the threshold inducing spontaneous polarization in a homogeneous pheromone concentration). Two additional elements are required for the model to achieve its predictive power: temporal evolution and non-linear spatial interactions between different parts of the cell. These elements appear in the equations that make the core of the model. The experiments in homogeneous pheromone concentration allowed us to fit the values of the parameters that determined the appropriate dynamical and non-linear behavior (namely the spatial correlation length *χ* and the Cdc42 endocytosis rate *k*_off_) and the cell-to-cell variability (namely *δ*). Due to the non-linear and non-local aspects of the model, it is very sensitive to these parameter values. As a consequence, they could be determined precisely from the experimental data, giving the model its predictive power. Interestingly, although we did not study the details of the dynamical aspects of the polarization process, the model reproduces many features of this dynamics (Fig 4E and 4F). In the model, membrane associated Cdc42 forms moving patches with several large patches at initial time point (at 1h20 and 2h20) and eventually forms the single patch that stops moving and becomes gradually more focused (Fig 4E). The typical behavior of Spa2 protein localization follows qualitatively similar dynamics. Spa2 appears as weak dispersed and moving multiple spots that eventually coalesce into one spot that stops moving and accumulates more signal (note that because the total protein content being conserved during the simulation there is also a higher density when the spot becomes more focused (at 6h20)). Multiple small Spa2 spots observed early in the polarization process might correspond to a weak polarization of Cdc42 which was not directly studied in this work. The rotation of the polar cap was also reported by [17]. Although our model also shows initial rotation of the polar cap we did not specifically study this process.

The finding that cells accurately sense the gradient in an extremely narrow concentration range is intriguing. This finding appears to contradict the expectations for gradient sensing in vivo, where the presence of many different pheromone secreting cells will produce a complex concentration landscape fluctuating across a larger range of concentrations than the narrow window suggested by the model. But previous studies [56, 57] suggest that, thanks to the screening effects of the Bar1 protease, cells only sense their closest neighbors, and the ability of Bar1 to regulate the fraction of local pheromone molecules that reach the receptors could produce a narrow range of effective pheromone concentrations at the cell surface.

Our model describes spontaneous polarization in an homogeneous pheromone concentration, starting from a homogeneous initial internal state, see [25–27]. In addition to the homogeneous initial distribution, we tested several different initial conditions: from totally random initial Cdc42 concentration to Gaussian variation in the initial Cdc42 concentration. Although we could obtain results in good agreement with experimental data by using Gaussian initial conditions with particular choice of the variance (Fig G in S1 Text), we decided not to use this approach since it involved parameters that we were not able to estimate. Instead we added stochasticity to the parameter *κ*, which describes the dynamics of the effective pheromone receptors. This had the advantage of accounting for both the initial conditions and noise in the signalling pathway with a minimal number of parameters. One of these parameters, the damping coefficient λ in the noise (1/λ corresponds to the mean time to reach the steady state), had a crucial effect on the model output when considering a very large set of values (Fig H in S1 Text), but a weak effect when considering variations in a realistic range of values (Fig 4D). When λ was too large (the steady state is reached very fast which is equivalent with removing the stochasticity), the direction of polarization was always more accurate than what was experimentally observed. On the contrary, when λ was too small (the steady state is never reached), the direction of polarization was never accurate with respect to the gradient. The range of λ values that produced the good agreement between experiment and prediction was estimated based on physical arguments. Indeed, 1/λ can be interpreted as the fastest timescale in the biochemical signalling pathway, hence its mean value must lie between seconds and minutes, a range of values which gives the correct model prediction for gradient sensing (we found that the optimal value for 1/λ was 3 mn (Fig 4D)). Such an optimal value is not very different from the value 1/λ = 10 mn which was used in the numerical simulations. Similarly to λ the parameter *N*, which specifies the number of independent membrane domains, had a weak effect on the model output in a realistic range of values (from 5 to 20 corresponding to the cell perimeter divided by the polarisome size).

The model we propose has the advantage of simplicity. Although it involves sixteen parameters, only three of them were unknowns, the spatial correlation length *χ*, the Cdc42 endocytosis rate *k*_off_ and the cell-to-cell variability, *δ*. The cell-to-cell variability, was fitted with the standard deviation in the delay for polarization in uniform pheromone concentration. The two remaining parameters, *χ* and *k*_off_, were fitted with the data in uniform pheromone concentration. These two latter parameters represent the two principal aspects that control the model: *χ* for the long-range spatial correlation and the endocytosis rate *k*_off_ for the reaction/diffusion phenomena. If we vary these two key parameters from the values that we get by fitting the behavior of cells in uniform pheromone, we lose the fit for the gradient response data. Indeed, for values of *χ* and *k*_off_ away from the optimum, polarization will either polarize too fast or too slow. If polarization is too fast, accuracy in gradient detection is lost, if polarization occurs too slow gradient detection is better that what is observed experimentally. This shows that the strong constraint on parameter choice obtained from in real data cells polarizing in uniform field of pheromone makes the model predictive enough to fit the gradient response data. It would be interesting, in future studies, to test if mutants in gradient detection also show a shift in the parameters obtained from experiments of polarization in uniform pheromone fields.

Our model does not explicitly take into account the phenomenon of receptor polarization. Indeed, at the timescales at which polarization is first observed (one hour or more Fig G in S1 Text), receptor polarization has likely already occurred (receptor polarization within 30–60 minutes after pheromone treatment is reported in [58] e.g.). Our model suggests that long-range spatial coupling is critical in the gradient detection process, but at the molecular level, many different mechanisms could produce this coupling including the transport and recycling of the pheromone receptor itself. The model’s strength is its indepedence from the details of the molecular mechanisms that determine the long-range coupling parameter. The cost of this simplicity is that the model will not distinguish different mutants that affect the same parameter. In the future the model could be enriched with molecular details provided experimental data are available to fit the additional parameters.

From a mathematical viewpoint, eqs (1) and (2) are very close to the Keller-Segel model for chemotaxis. This model has been mathematically studied for four decades due to its dichotomous behavior between an aggregated state and a homogeneous one ([59–63]). The main difference between the Keller-Segel model and our model is that in our case the source of the attractive potential (the pheromone induced array of actin filaments inside the cell) from which the positive feedback loop originates is supported by the boundary of the domain (i.e. the cell membrane), while in the Keller-Segel model the source of the chemical potential (the cells producing the attractant molecules) is distributed everywhere across the spatial domain (the space in which the cells move). Our model has been mathematically studied in [26, 27]. In the one dimensional case, its dynamics are well understood [64] and are reminiscent of the Keller-Segel model in two dimensions. In the more realistic two dimensional case, we used a numerical approach [55] and some heuristics [27] to show that the model has a two-state behavior which represents the two states of the cell: polarized or unpolarized. Numerical simulations, [55], show that for large enough values of *S*, the majority of the Cdc42 molecules are located in the neighborhood of the cell membrane, hence we postulate that ∫*n*(*t*,**x**)*d* **x**_∥_ = *μ*(*t*, *s*). Under this assumption we can formally write the dynamics of *μ*(*t*, *s*) (the Cdc42 concentration on the cell membrane) by integrating eq (1) with respect to the normal coordinate (to the membrane) and we obtain a one dimensional, convection diffusion equation in which the advective field is given by a non-local operator (long-range spatial coupling between sites on the membrane is modeled by a non-local positive feedback). In the particular case of a two dimensional model, this operator is similar to the Hilbert transform H (see S1 Text). The one-dimensional non-local convection-diffusion equation is then

$$\begin{array}{c} \partial_{t}\mu =D_{b}\partial_{ss}\mu +\chi \;\partial_{s}\left(\mu H\left(\kappa \frac{S}{S_{0}+S}\mu \right)\right)\;. \end{array}$$ (3)

This equation is known to have a solution which aggregates in a finite time if *χ∫μ* (the total membrane Cdc42 mass) is large enough as compared to the values of *κ* and *S*, see [65]. In our setting, this analysis means that for small values of the total mass, ∫*μ*, the cell remains unpolarized while for large values it gets polarized (see Fig 3A and 3B). Investigating the spatial heterogeneity of the pheromone receptor activity *κ* and the pheromone concentration *S* (the stochasticity of the celullar response and the pheromone concentration) is one contribution of the present work.

We believe that the dichotomous behavior of the model which was observed in a two dimensional setting would also appear in a 3D framework. Indeed as it is the case for the Keller-Segel equation, the 3D case is expected to more easily lead to polarization than the 2D case. Finally, since the model, defined by the eqs (1) and (2), is deterministic, a mathematical challenge is to establish that these equations can be derived as the mean field limit of microscopic stochastic processes. We leave such a justification for further work.

While our phenomenological approach has the advantage of providing a fully fitted predictive model from a minimal set of experimental data, it does not account for many known biological processes which might affect polarization in the context of the real cell-cell communication. The main outputs of the model are the dynamics and directionality of polarization in a stationary pheromone concentration field. During processes involving cell-cell communication, such as mating, the pheromone concentration field might vary in time. An important task will be to add to this model a description of the dynamics of the signaling pathway in response to these temporal fluctuations.

We propose that, like the capacity of Keller-Segel equations to describe cell aggregation behaviors associated with chemotaxis, our model constitutes a phenomenologically minimal framework to describe the formation of local regions of increased protein concentrations associated with cell polarization in response to external signals.

## Methods

This is a skeletal description; a more detailed one is found in S1 Text.

### Yeast strains

Standard yeast manipulation methods were used. Strains used in this study are listed in Table A in S1 Text. All the strains constructed in this study are in the W303 background unless otherwise specified.

### Microfluidic chambers

The design and fabrication of the chambers was based on standard soft lithography techniques. Cells were bound to the chambers by cross-linking Concanavalin A, a lectin, which strongly binds to the yeast cell wall, to the coverslips that formed the roof of the chambers. The chambers and tubing were pre-coated with BSA. When several strains were to be studied in parallel in a given chamber, they were differentially stained prior to binding to the chamber by covalently labeling their cell walls N-hydroxy succinimide esters of different fluorescent dyes. The two inlets to the chamber were coupled to two reservoirs: one containing complete synthetic growth medium and the other containing the same growth medium plus the desired concentration of *α*-factor and 200 *μ*g/ml of Texas Red conjugated dextran (MW 3000, Molecular Probes). Using the fluorescent dextran in the pheromone flow, the flows were adjusted to be equal at the point where they first encountered each other in the chamber (Fig A in S1 Text). We recorded the fluorescent dextran profile (as well as cellular behavior), both to calculate the local pheromone concentration and to check the temporal and spatial stability of the *α*-factor concentration during the experiment (Fig C in S1 Text).

### Image processing and analysis

Cell behavior was analyzed semi-automatically. Interactive scripts facilitated the analysis and recording of various aspects of the behavior of each cell. For promoter activity, we measured the total intensity in the YFP image from each cell. The total YFP intensity in cells treated with a constant level of pheromone would increase linearly for hours and the slope was measured to obtain the promoter activity for individual cells. Images of the fluorescent dextran were used to estimate the *α*-factor concentration.

## Supporting Information

S1 Text Supporting Information. (PDF)
