# Supplementary material for: A Predictive Model for Yeast Cell Polarization in Pheromone Gradients
Source: PLoS Comput Biol. 2016 Apr 14;12(4):e1004795. doi: 10.1371/journal.pcbi.1004795 (PMC4831791; doi:10.1371/journal.pcbi.1004795)

A predictive model for yeast cell polarization in pheromone gradients - Supplementary material

Nicolas Muller[1,◑], Matthieu Piel[2,*,◑], Vincent Calvez[3], Raphaël Voituriez[4], Joana Gonçalves-Sá[5], Chin-Lin Guo[5,6], Xingyu Jiang[7,8], Andrew Murray[5,*], Nicolas Meunier[1,*]

1 MAP5, CNRS UMR 8145, Université Paris Descartes, 45 rue des Saints-Pères, 75270 Paris Cedex 06, France
2 Institut Curie, CNRS UMR 144, 26 rue d'Ulm 75248 Paris Cedex 05, France
3 Unité de Mathématiques Pures et Appliquées, CNRS UMR 5669, & équipe-projet INRIA NUMED, École Normale Supérieure de Lyon, 46 allée d'Italie, F-69364 Lyon, France
4 Laboratoire Jean Perrin & Laboratoire de Physique Théorique de la Matière Condensée, UMR 7600 CNRS /UPMC, 4 Place Jussieu, 75255 Paris, France
5 Molecular and Cell Biology & FAS Center for Systems Biology, Harvard University, 52 Oxford St., Cambridge, MA 02138, USA
6 Bioengineering and Applied Physics, California Institute of Technology, MS 138-78, 1200 East California Boulevard, Pasadena, CA 91125, USA
7 Department of Chemistry and Chemical Biology, Harvard University, 12 Oxford Street, Cambridge, MA 02138 , USA
8 CAS Key Laboratory for Biological Effects of Nanomaterials and Nanosafety, National Center for Nanoscience and Technology, Beijing 100190, PR China

◑These authors contributed equally to this work.
* matthieu.piel@curie.fr **and** nicolas.meunier@parisdescartes.fr **and** amurray@mcb.harvard.edu

1 Computational Methods

1.1 Model description

We assume that $\Omega = B(O,R) \setminus B(O,R_{\mathrm{n}})$ describes the cell, thus the cell periphery is the circle of radius R. The cytoplasmic Cdc42 density is denoted by $n(t,\mathbf{x})$, the membrane density of Cdc42 is denoted $\mu(t,s)$ and the density of cytoskeletal tracks is denoted by $c(t,\mathbf{x})$. Let us recall the model

$$\begin{cases} \partial_t n(t,\mathbf{x}) = D_b\,\Delta n(t,\mathbf{x}) - \chi\,\nabla\cdot\Big(n(t,\mathbf{x})\,\nabla c(t,\mathbf{x})\Big) & \text{if } R_n < \|\mathbf{x}\| < R\,, \\ \partial_t \mu(t,s) = D_m\,\partial_{ss}\mu(t,s) + k_{\mathrm{on}}\,n(t,s) - k_{\mathrm{off}}\,\mu(t,s) & \text{if } \|\mathbf{x}\| = R\,, \end{cases} \tag{1}$$

where s denotes the curvilinear abscissa associated with the point $\mathbf{x}\in\partial\Omega$ such that $\|\mathbf{x}\| = R$, k_{on} and k_{off} are the attachment and detachment rates of Cdc42 to and from the membrane.

This system is complemented by an initial condition $(n_0(\mathbf{x}), \mu_0(s), c_0(\mathbf{x}))$.

Furthermore, the conservation of total protein pool, defined by

$$M := \iint_{\Omega} n_0(\mathbf{x}) d\mathbf{x} + \int_{\|\mathbf{x}\|=R} \mu_0(s) ds\,, \tag{2}$$

is guaranteed by a zero flux boundary condition on the cell nucleus, $C(O, R_n)$, and by an additional boundary condition on the cell membrane:

$$\Big(D_b \nabla n(t,s) - \chi\, n(t,s)\, \nabla c(t,s) \Big) \cdot \vec{e}_{\mathbf{x}} = -\partial_t \mu(t,s) \quad \text{on } C(O,R)\,, \tag{3}$$

where $\vec{e}_{\mathbf{x}}$ is the outward unit normal vector to Ω at point $\mathbf{x} \in \partial\Omega$ such that $\|\mathbf{x}\| = R$ of curvilinear abscissa s. The density of cytoskeletal tracks, c, which is involved in the active transport, with the advection field $\chi \nabla c$, and which accounts for a positive feedback loop mediated by actin-based transport, (Figure 1C), satisfies:

$$\begin{cases} -\Delta\, c(t,\mathbf{x}) + \eta\, c(t,\mathbf{x}) = 0 \quad \text{on } \Omega\,, \\ -\nabla c(t,s) \cdot \vec{e}_{\mathbf{x}} = \kappa\, \frac{S(s)}{S_0 + S(s)}\, \mu(t,s) \quad \text{on } C(O,R)\,. \end{cases} \tag{4}$$

We note that changing c into c/κ in (4) leads to multiply the advection field by κ in (3). Hence the value of κ can be fixed, we choose $\kappa = 13$ as it will be explained below.

1.2 Ornstein-Uhlenbeck process to describe the evolution of κ

Let us now describe the purely phenomenological model whose actors have no direct relationship to the molecules known to be responsible for pheromone signaling which was used to model the processes that take place once the pheromone binds some receptor molecule on the cell membrane.

In order to describe the noise in the signalling pathway we added stochasticity to the parameter κ, which describes the dynamics of the pheromone receptors. More precisely, we assumed that $\kappa(t,\mathbf{x})$ is a space-time stochastic process, of Ornstein-Uhlenbeck type. The choice of an Ornstein-Uhlenbeck process was motivated by the fact that cell polarization is a dynamical process and that the Ornstein-Uhlenbeck process is the simplest Gaussian process of order 2.

To incorporate spatial correlation in κ, we broke the cell membrane into N different sectors (Figure 1D). On each of the N parts, $\mathbf{x} \to \kappa(t,\mathbf{x})$ was supposed to be a constant function, i.e. $\kappa(t,\mathbf{x}) = \kappa^i(t)$ for all $\mathbf{x} \in \Gamma_i$ with $\Gamma_i \subset \Gamma$, $|\Gamma_i| = |\Gamma|/N = 2\pi R/N$. Finally, for all $i \in \{1, \ldots, N\}$, κ^i is an Ornstein-Uhlenbeck random process $(\kappa^i_t)_{t\geq 0}$ defined by

$$d\kappa^i_t = -\lambda \left(\kappa^i_t - \tilde{\kappa}_{\text{mean}} \right) dt + \sigma dW_t\,, \tag{5}$$

where $(W_t)_{t\geq 0}$ is a standard Brownian motion, λ is a damping coefficient, σ is the standard deviation of the instantaneous change (Figure 1 E) and $\tilde{\kappa}_{\text{mean}}$ will be defined in the next subsection. The damping coefficient reflects the combined action of pheromone binding and unbinding, and the lateral diffusion and endocytosis of the receptor.

Moreover, as is usual when counting a number of independent events in a specified interval, we assume that the initial number of receptors κ^i_0 located on Γ_i has a Poisson distribution with mean κ_{mean}.

Furthermore, we impose the moments of the noise to be stationary. Indeed, the noise must be independent of the system, it would not make any sense to consider a process that would have two different dynamics (i.e. have different moments) at time t and at time $t + \Delta t$. Imposing the moments of the noise to be stationary is similar to assume the following relationship between the two parameters σ and λ, see [1],

$$\frac{\sigma^2}{2\lambda} = \kappa_{\text{mean}}$$

1.3 Cell-to-cell variability

In addition to the previous source in noise in the pheromone receptors activity, κ, whose variation represents noise in both space and time within a single cell, we consider a second source of noise to describe the cell-to-cell variability in the total Cdc42 pool M. Indeed the value of the total Cdc42 pool might fluctuate between cells.

From the mathematical viewpoint, a variation of M is similar to a variation of $\tilde{\kappa}_{\text{mean}}$. Thus we assumed $\tilde{\kappa}_{\text{mean}}$ to be a random variable that has a normal distribution with mean κ_{mean} and variance δ.

If we remove the cell-to-cell variability in the model, we observe that the model fits with the sigmoidal data but less precisely (Fig F in S1 Text). This suggests that the total number of proteins, M, can not be considered as fixed for all cells.

1.4 Only three parameters remain free.

All in all, the model involves sixteen parameters, see Table 1: D_b, D_m, M, k_{on}, k_{off}, the mean value κ_{mean}, the spatial correlation $2\pi R/N$, the cell-to-cell variability δ, χ, η, R, R_{n}, S_0, λ, σ and the initial condition $(n_0(\mathbf{x}), \mu_0(\mathbf{x}), c_0(\mathbf{x}))$. Six parameters are available from the literature: the cytoplasmic diffusion coefficient $D_b \sim 0.02\,\mu\text{m}^{\,2}/s$, the cell radius $R \sim 2.5\,\mu\text{m}$, [2], the nuclear radius $R_{\text{n}} \sim 1\,\mu$ m, the total molecular content $M \sim 10^3$, see [3], the actin depolymerisation rate $\eta = 10^{-3}s^{-1}$ [4], and the K_d of pheromone receptor $S_0 \sim 6$ nM, [5]. The influence of the parameters R_{n} and η are negligible.

The observed size of a polarisome which occupies 10% of the total membrane length, see [3], fixes the correlation length of the noise, numerically this is similar to fixing the number $N \sim 10$ of sectors. Since only the product $\chi\kappa$ affects the solution of the model, the value of κ_{mean} can be fixed arbitrarely, we choose $\kappa_{\text{mean}} = 13$ in order the following condition to be satisfied.

The observation that, without any pheromone, 13% of the Cdc42 molecules are on the membrane at steady state, see [3,6], fixes the value of the ratio $k_{\text{on}}/k_{\text{off}}$. Indeed for $S = 0$, the stationary state of the system (1)-(4) is solution of

$$\begin{aligned} D\Delta n^*(\mathbf{x}) &= 0\,, \qquad \mathbf{x} \in \Omega, \\ D_m \Delta\mu^*(\theta) + k_{\text{on}}\rho^*(R,\theta) - k_{\text{off}}\,\mu^*(\theta) &= 0\,, \qquad \theta \in \mathbb{R}/2\pi\mathbb{Z}, \\ \int_\Omega \rho^*(\mathbf{x})\,d\mathbf{x} + \int_\Gamma \mu^*(\mathbf{x})\,d\mathbf{x} &= M. \end{aligned} \tag{6}$$

The stationary solution K is the unique solution of the system (6). Hence, on the cell membrane, we have

$$n^*(R,\theta) = \frac{k_{\text{off}}}{k_{\text{on}}}\mu^*(\theta) = K, \quad \theta \in \mathbb{R}/2\pi\mathbb{Z}.$$

Recalling that $|\Omega| = \pi\left(R^2 - R_{\text{n}}^2\right)$ and $|\Gamma| = 2\,\pi\,R$, and using the conservation of the total protein pool we obtain that

$$\begin{aligned} \int_\Omega n^*(\mathbf{x})\,d\mathbf{x} &= \pi\,(R^2 - R_{\text{n}}^2)\,K = 87\%\,M, \\ \int_\Gamma \mu^*(\mathbf{x})\,d\mathbf{x} &= 2\,\pi\,R\,\frac{k_{\text{on}}}{k_{\text{off}}}\,K = 13\%\,M. \end{aligned}$$

Consequently,

$$\frac{k_{\text{on}}}{k_{\text{off}}} = \frac{13}{2\times 87\times R}\,(R^2 - R_{\text{n}}^2) = 0.16\mu\text{m}.$$

Notice that traditional k_{off} has units s^{-1}, and k_{on} has units $M^{-1}s^{-1}$ meaning that the ratio should have units M^{-1}. Here the ratio is a linear distance as a consequence of redefining units in equation (4). As a consequence, k_{on} and k_{off} are effective dissociation and association constants.

Furthermore, we assume that the range of action in the reaction-diffusion equation on the membrane (1) is similar to the one considered in [3], i.e. $\sqrt{D_m/k_{\text{off}}} \sim 0.37\,\mu$m. Finally, in order the processes $\left(\kappa_t^i\right)_t$ to have a stationary intensity, i.e. $Var(\kappa_t) = 0$, we impose $\sigma^2/2\lambda$ to be equal to the variance of the Poisson distribution κ_0^i.

The damping coefficient λ reflects the combined action of pheromone binding and unbinding, and the lateral diffusion and endocytosis of the receptor. It is therefore dominated by the fastest of these processes. Physical arguments provide bounds for $1/\lambda$, which corresponds to a relaxation delay. At high pheromone concentration, this delay is the half time for endocytosis, that is on the order of a minute, [7], hence we suppose that $1/\lambda \sim 1$ min.

In the experiments described above, cells were suddenly exposed, at time zero, to a wide range of temporally stable concentrations of pheromone. Therefore, in our numerical experiments we assumed an initial distribution of Cdc42 corresponding to a steady state pheromone concentration, $S = 0$ nM, at the cell membrane. Hence we considered that the initial condition is given by (6) which corresponds to the assumption that initially 13% of the Cdc42 molecules were on the membrane, see [3, 6].

In conclusion, as it is explained above, except three parameters, χ, k_{off} and δ, the values of all parameter involved in the model can be either found in the literature or computed by using biological, physical or mathematical arguments.

1.5 Dimensioning the model

As it is classical we make a dimensional analysis. Let us consider the following typical quantities for nondimensionalization

$$\mathbf{x}' = \frac{\mathbf{x}}{R_{\text{n}}},\ t' = \frac{t}{\bar{t}},\ n' = \frac{n\,R_{\text{n}}^2}{M},\ \mu' = \frac{\mu\,R_{\text{n}}}{M},\ c' = \frac{c\,R_{\text{n}}^2}{M D_b},\ \kappa' = \frac{\kappa\,R_{\text{n}}^2}{D_b}\,.$$

Hence the nondimensionalized problem associated to the equations (1–4) now writes

$$\partial_{t'} n'(t',\mathbf{x}') = \left(\frac{D_b\,\bar{t}}{R_{\text{n}}^2}\right)\Delta_{\mathbf{x}'} n'(t',\mathbf{x}') - \left(\frac{\chi M \bar{t}}{\kappa R_{\text{n}}^4}\right)\nabla_{\mathbf{x}'}\cdot\left(n'(t',\mathbf{x}')\nabla_{\mathbf{x}'} c'(t',\mathbf{x}')\right)\,,\quad \mathbf{x}'\in\Omega', \tag{7}$$

$$\partial_{t'}\mu'(t',\theta) = \left(\frac{D_m\,\bar{t}}{R_{\text{n}}^2}\right)\partial_{\theta\theta}\mu'(t',\theta) + \left(\frac{k_{\text{on}}\,\bar{t}}{R_{\text{n}}}\right) n'(t',R',\theta) - \left(k_{\text{off}}\,\bar{t}\right)\mu'(t',\theta)\,,\quad \theta\in\mathbb{R}/2\pi\mathbb{Z}, \tag{8}$$

$$-\Delta_{\mathbf{x}'} c'(t',\mathbf{x}') + \left(\eta\,R_{\text{n}}^2\right) c'(t',\mathbf{x}') = 0\,,\qquad \mathbf{x}'\in\Omega', \tag{9}$$

$$\nabla c'(t',\theta).\vec{n}_\theta = \kappa'\frac{S(\theta)}{S_0 + S(\theta)}\,\mu'(t',\theta)\,,\qquad \theta\in\mathbb{R}/2\pi\mathbb{Z}, \tag{10}$$

$$\int_{\Omega'} n'(t',\mathbf{x}')\,d\mathbf{x}' + \int_{\Gamma'}\mu'(t',\mathbf{x}')\,d\mathbf{x}' = 1, \tag{11}$$

with $\Omega' = B(0,2.5)\setminus B(0,1)$ and $\Gamma' = C(0,2.5)$, according to the values of R and R_{n}.

1.6 Numerical polarization procedure

We first describe the numerical criterion for polarization. Using the image (Fig B in S1 Text) we observe that the mean length of a shmoo is around 1/3 of the cell diameter (Fig G in S1 Text). This allows defining the mean size of the shmoo: $\dfrac{2\,R}{3} \simeq 1.66\,\mu m$,

which represents 10% of the cell perimeter Γ. This observation is in good agreement with the polarization criterion used in [3]: *"Polarization in simulations was determined by whether an interval of* 10% *of the membrane contained more than* 50% *of total membrane content"*. Consequently we consider this criterion which we formulate as following.

A cell is polarized in x_0 at time T if there exists a connex part $\Gamma_{\rm pol}$ of Γ of size $10\%\,|\Gamma|$ containing x_0 such that for all $t \geq T$

$$\int_{\Gamma_{\rm pol}} \mu(t,s)\,ds \geq \frac{1}{2}\int_{\Gamma} \mu(t,s)\,ds\,.$$

We adapt this definition to obtain a numerical criterion by using the discretization $(\mu_k^n)_{1\leq k\leq N_\theta}$ of μ. In all our simulations, the location of polarization and the timing of polarization are defined as following: We consider that the cell is polarized in $\theta_j = j\,\Delta\theta \in \mathbb{R}/2\pi\mathbb{Z}$, $j \in \{1,...,N_\theta\}$ if there exists a time T such that for all $t^n = n\,\Delta t \geq T$ the following conditions are verified

$$\sum_{k=j-\lfloor\frac{N_\theta}{5}\rfloor}^{j+\lfloor\frac{N_\theta}{5}\rfloor} \mu_k^n \geq \frac{1}{2}\sum_{k=1}^{N_\theta}\mu_k^n \quad \text{and} \quad \mu_j^n = \max_{k\in\{1,\ldots,N_\theta\}} \mu_k^n\,.$$

1.7 Optimization procedure

First, we observed that if $\log(k_{\rm off}) \leq -2.5$, polarisomes can not stabilize (Figure 3C). Next, noting that the transformation $(S,\chi,k_{\rm off}) \to (1, \chi\frac{S_0+S}{S}\frac{1}{S_0+1}, k_{\rm off})$ leaves the solution (n,μ) of the problem invariant, we can determine the optimal couple $(\chi, k_{\rm off})$ by interpolating the polarization delay curve between $S = 1$ nM and $S = 10$ nM (hence for a fixed value of $k_{\rm off}$, a translation to the left of (Fig. 3C) of $\log\left(\frac{S_0+S}{S}\frac{1}{S_0+1}\right)$ provides the isovalues of the mean polarization delay for a given value S as a function of $(\chi, k_{\rm off})$ (Figure 3D)). The value $\delta = 0.2$ was then found by a second optimization procedure with the standard deviation of the polarization delay in uniform pheromone concentration.

1.8 Sensitivity to the initial condition

In the biological experiments, the initial Cdc42 concentration is unknown. We have tested the situation where the initial condition is a Gaussian function and we have considered several variances. More precisely, we have considered that the initial distribution of Cdc42 is a Gaussian centered in $\mathbf{x}_0 \in \Gamma$ whose standard deviation ω took its value between 10 and 50:

$$\begin{cases} n_0(\mathbf{x}) = \exp\left(-\dfrac{||\mathbf{x}-\mathbf{x}_0||^2}{\omega^2}\right), & \mathbf{x}\in\Omega, \\ \mu_0(\theta) = \dfrac{k_{on}}{k_{off}}\,\rho_0(R,\theta), & \theta \in \mathbb{R}/2\pi\mathbb{Z}. \end{cases}$$

For every mean pheromone concentration S_{mean} and for all standard deviation, we numerically solve the equations for 8 initial conditions with $\mathbf{x}_0 = (R_{max}, \theta_0)$ and $\theta_0 \in \{\frac{k\,\pi}{4}$ with $k \in \mathbb{Z}\}$.

The higher the standard deviation is, the lower the proteins are concentrated and hence the advection field is locally less strong. The cell will take more time to polarize.

On the opposite the smaller the standard deviation is the faster the cell will polarize in a neighbourhood of $\mathbf{x}_0$. In S1 Text, Fig G we see that the numerical results corresponding to $\omega = 40$ are in good agreement with the experimental data. However, we did not consider such an initial condition since it involves some parameters that we were not able to fit.

1.9 Numerical scheme

For simplicity, in this section we take all constants equal to 1 except η which is very small. Since the domain Ω is an annulus, it is appropriate to introduce polar coordinates r and θ.

Let $\mathbf{x} = (r\cos\theta, r\sin\theta) \in \Omega$ and let $\tilde{n}$ be defined by $\frac{1}{r}\tilde{n}(t,r,\theta) = n(t,\mathbf{x})$ with $(r,\theta) \in [R_{\mathrm{n}}, R] \times \mathbb{R}/2\pi\mathbb{Z}$, we have the following equations:

$$\begin{aligned}
\partial_t \tilde{n}(t,r,\theta) &= \partial_r \left(r\partial_r \left(\frac{\tilde{n}(t,r,\theta)}{r} \right) - \tilde{n}(t,r,\theta)\mathbf{u}_r(t,r,\theta) \right) \\
&+ \partial_\theta \left(\frac{1}{r^2} (\partial_\theta \tilde{n}(t,r,\theta) - \tilde{n}(t,r,\theta)\mathbf{u}_\theta(t,r,\theta)) \right) \text{ in } \Omega, \qquad (12) \\
-\partial_t \mu(t,\theta) &= r\partial_r \left(\frac{\tilde{n}(t,R,\theta)}{r} \right) - \tilde{n}(t,R,\theta)\mathbf{u}_r(t,R,\theta), \text{ on } C(O,R), \qquad (13) \\
0 &= R_{\mathrm{n}}\partial_r \left(\frac{\tilde{n}(t,R_{\mathrm{n}},\theta)}{r} \right) - \tilde{n}(t,R_{\mathrm{n}},\theta)\mathbf{u}_r(t,R_{\mathrm{n}},\theta), \text{ on } C(O,R_{\mathrm{n}}). \qquad (14)
\end{aligned}$$

Let t be fixed, setting $\frac{1}{r}\tilde{c}(r,\theta) = c(\mathbf{x})$ with $(r,\theta) \in [R_{\mathrm{n}}, R] \times \mathbb{R}/2\pi\mathbb{Z}$, we also give the equations in polar coordinates for $\tilde{c}$:

$$-\partial_r \left(r\partial_r \left(\tfrac{\tilde{c}(r,\theta)}{r} \right) \right) - \tfrac{1}{r^2}\partial_{\theta\theta}\tilde{c}(r,\theta) + \eta\,\tilde{c}(r,\theta) = 0, \text{ in } \Omega, \qquad (15)$$

$$\partial_r \left(\tfrac{\tilde{c}(r,\theta)}{r} \right) = \mu(t,\theta), \text{ on } C(O,R), \qquad (16)$$

$$\partial_r \left(\tfrac{\tilde{c}(r,\theta)}{r} \right) = 0, \text{ on } C(O,R_{\mathrm{n}}). \qquad (17)$$

Let $t^n = n\,\Delta t$ be the time discretization and $\{r_j = R_{\mathrm{n}} + j\,\Delta r, j \in \{1,...,N_r\}\}$ (resp. $\{\theta_k = k\,\Delta\theta, k \in \{1,...,N_\theta\}\}$) be the space discretization of the bounded interval $[R_{\mathrm{n}}, R]$ (resp. periodic interval $\mathbb{R}/2\pi\mathbb{Z}$), where $\Delta t = 10^{-2}$ and$\Delta r = \Delta\theta = \dfrac{2\pi}{120}$. We introduce the control 2D "volume" $W_{(j,k)} \subset \mathbb{R}^2$

$$W_{(j,k)} = \left(r_{j-\frac{1}{2}}, r_{j+\frac{1}{2}} \right) \times \left(\theta_{k-\frac{1}{2}}, \theta_{k+\frac{1}{2}} \right).$$

Let $\tilde{P}^n_{(j,k)}$ (resp. μ^n_k) be the approximated value of the exact solution $\tilde{n}(t^n, r_j, \theta_k)$ (resp. $\mu(t^n,\theta_k)$). Let $\tilde{c}_{(j,k)}$ be the approximated value of the exact solution $\tilde{c}(r_j,\theta_k)$.

1.10 Equation on $\tilde{c}$

If, for simplicity, we call $\mathcal{F}$ the numerical flux, we can write the following scheme for equation (15) for $(j,k) \in \{1,...,N_r\} \times \{1,...,N_\theta\}$

$$-\left(\frac{\mathcal{F}_{(j+\frac{1}{2},k)} - \mathcal{F}_{(j-\frac{1}{2},k)}}{\Delta r} + \frac{\mathcal{F}_{(j,k+\frac{1}{2})} - \mathcal{F}_{(j,k-\frac{1}{2})}}{\Delta\theta} \right) + \eta\,\tilde{c}_{(j,k)} = 0,$$

where

$$\mathcal{F}_{(j+\frac{1}{2},k)} = r_{j+\frac{1}{2}} \frac{\frac{\tilde{c}_{(j+1,k)}}{r_{j+1}} - \frac{\tilde{c}_{(j,k)}}{r_j}}{\Delta r}, \qquad \mathcal{F}_{(j-\frac{1}{2},k)} = r_{j-\frac{1}{2}} \frac{\frac{\tilde{c}_{(j,k)}}{r_j} - \frac{\tilde{c}_{(j-1,k)}}{r_{j-1}}}{\Delta r},$$

$$\mathcal{F}_{(j,k+\frac{1}{2})} = \frac{1}{r_j^2} \frac{\tilde{c}_{(j,k+1)} - \tilde{c}_{(j,k)}}{\Delta\theta}, \qquad \mathcal{F}_{(j,k-\frac{1}{2})} = \frac{1}{r_j^2} \frac{\tilde{c}_{(j,k)} - \tilde{c}_{(j,k-1)}}{\Delta\theta}.$$

The zero flux boundary condition (17) imposes that $\mathcal{F}_{(\frac{1}{2},k)} = 0$ and the boundary condition (16) imposes that $\mathcal{F}_{(N_r+\frac{1}{2},k)} = r_{N_r+\frac{1}{2}}\,\mu_k^n$ for $k \in \{1, ..., N_\theta\}$. Similarly, for $j \in \{1, ..., N_r\}$, the periodic conditions impose

$$\mathcal{F}_{(j,N_\theta+\frac{1}{2})} = \mathcal{F}_{(j,\frac{1}{2})} = \frac{1}{r_j^2}\frac{\tilde{c}_{(j,1)} - \tilde{c}_{(j,N_\theta)}}{\Delta\theta}.$$

We define the column vector

$$\mathcal{C} = \left(\tilde{c}_{(1,1)} \,\dots\, \tilde{c}_{(1,N_\theta)}\, \tilde{c}_{(2,1)} \,\dots\, \tilde{c}_{(2,N_\theta)} \,\dots\, \tilde{c}_{(N_r,N_\theta)}\right)^T.$$

For $\Delta r = \Delta\theta$ the rigidity matrix $\mathcal{A}$ is defined by

$$\mathcal{A} = \begin{pmatrix} \ddots & \ddots & \ddots & & \\ & -\frac{r_{j-\frac{1}{2}}}{r_{j-1}} Id & \frac{r_{j-\frac{1}{2}}+r_{j+\frac{1}{2}}}{r_j} Id & -\frac{r_{j+\frac{1}{2}}}{r_{j+1}} Id & \\ & & \ddots & \ddots & \ddots \end{pmatrix} + \begin{pmatrix} \frac{1}{r_1^2}A & & & & \\ & \frac{1}{r_2^2}A & & & \\ & & \ddots & & \\ & & & \frac{1}{r_{N_r-1}^2}A & \\ & & & & \frac{1}{r_{N_r}^2}A \end{pmatrix}. \tag{18}$$

From the flux boundary condition on $C(O,R)$ we deduce that the right hand side column vector of length $N_r\,N_\theta$:

$$\mathcal{R}_c^n = r_{N_r+\frac{1}{2}} \begin{pmatrix} 0 \\ \vdots \\ 0 \\ (\mu_k^n)_k \end{pmatrix}.$$

We use a standard numerical method to invert the symmetric positive definite matrix $\frac{1}{\Delta r^2}\mathcal{A} + \eta I_{N_r N_\theta}$. Then, at each time step, we solve

$$\mathcal{C} = \left(\frac{1}{\Delta r^2}\mathcal{A} + \eta I_{N_r N_\theta}\right)^{-1} \frac{1}{\Delta r}\mathcal{R}_c^n.$$

1.11 Equation on $\tilde{n}$

For simplicity, let us call $\mathcal{F}$ the numerical flux. We can write the following scheme for equation (12): for $(j,k) \in \{1, ..., N_r\} \times \{1, ..., N_\theta\}$

$$\frac{\tilde{P}_{(j,k)}^{n+1} - \tilde{P}_{(j,k)}^n}{\Delta t} = \frac{\mathcal{F}_{(j+\frac{1}{2},k)} - \mathcal{F}_{(j-\frac{1}{2},k)}}{\Delta r} + \frac{\mathcal{F}_{(j,k+\frac{1}{2})} - \mathcal{F}_{(j,k-\frac{1}{2})}}{\Delta\theta},$$

where

$$\mathcal{F}_{(j+\frac{1}{2},k)} = r_{j+\frac{1}{2}} \frac{\frac{\tilde{P}^{n+1}_{(j+1,k)}}{r_{j+1}} - \frac{\tilde{P}^{n+1}_{(j,k)}}{r_j}}{\Delta r} - A^{up}\left(u^{n+1}_{(j+\frac{1}{2},k)}, \tilde{P}^{n+1}_{(j,k)}, \tilde{P}^{n+1}_{(j+1,k)}\right),$$
$$\mathcal{F}_{(j-\frac{1}{2},k)} = r_{j-\frac{1}{2}} \frac{\frac{\tilde{P}^{n+1}_{(j,k)}}{r_{j}} - \frac{\tilde{P}^{n+1}_{(j-1,k)}}{r_{j-1}}}{\Delta r} - A^{up}\left(u^{n+1}_{(j-\frac{1}{2},k)}, \tilde{P}^{n+1}_{(j-1,k)}, \tilde{P}^{n+1}_{(j,k)}\right),$$
$$\mathcal{F}_{(j,k+\frac{1}{2})} = \frac{1}{r_j^2}\left(\frac{\tilde{P}^{n+1}_{(j,k+1)} - \tilde{P}^{n+1}_{(j,k)}}{\Delta\theta} - A^{up}\left(u^{n+1}_{(j,k+\frac{1}{2})}, \tilde{P}^{n+1}_{(j,k)}, \tilde{P}^{n+1}_{(j,k+1)}\right)\right),$$
$$\mathcal{F}_{(j,k+\frac{1}{2})} = \frac{1}{r_j^2}\left(\frac{\tilde{P}^{n+1}_{(j,k)} - \tilde{P}^{n+1}_{(j,k-1)}}{\Delta\theta} - A^{up}\left(u^{n+1}_{(j,k-\frac{1}{2})}, \tilde{P}^{n+1}_{(j,k-1)}, \tilde{P}^{n+1}_{(j,k)}\right)\right),$$

with the advection numerical flux is given by

$$A^{up}(u, x_-, x_+) = \begin{cases} u\,x_-, & \text{if } u > 0, \\ u\,x_+, & \text{if } u < 0. \end{cases} \tag{19}$$

We denote by $\mathbf{u}_r = \partial_r\left(\frac{\tilde{c}}{r}\right)$ and $\mathbf{u}_\theta = \frac{1}{r}\partial_\theta \tilde{c}$ and at time t^n we define

$$u^n_{(j+\frac{1}{2},k)} = \frac{\frac{\tilde{c}_{(j+1,k)}}{r_{j+1}} - \frac{\tilde{c}_{(j,k)}}{r_j}}{\Delta r}, \qquad u^n_{(j-\frac{1}{2},k)} = \frac{\frac{\tilde{c}_{(j,k)}}{r_{j}} - \frac{\tilde{c}_{(j-1,k)}}{r_{j-1}}}{\Delta r},$$
$$u^n_{(j,k+\frac{1}{2})} = \frac{1}{r_j}\frac{\tilde{c}_{(j,k+1)} - \tilde{c}_{(j,k)}}{\Delta\theta}, \qquad u^n_{(j,k-\frac{1}{2})} = \frac{1}{r_j}\frac{\tilde{c}_{(j,k)} - \tilde{c}_{(j,k-1)}}{\Delta\theta}.$$

The zero flux boundary condition (14) imposes that $\mathcal{F}_{(\frac{1}{2},k)} = 0$. Since there is an exchange on the boundary, we have $\mathcal{F}_{(N_r+\frac{1}{2},k)} = -\frac{\mu_k^{n+1} - \mu_k^n}{\Delta t}$ for $k \in \{1, ..., N_\theta\}$. Similarly, for $j \in \{1, ..., N_r\}$, the periodic conditions impose that

$$\mathcal{F}_{(j,N_\theta+\frac{1}{2})} = \mathcal{F}_{(j,\frac{1}{2})} = \frac{1}{r_j^2}\left(\frac{\tilde{P}^{n+1}_{(j,1)} - \tilde{P}^{n+1}_{(j,N_\theta)}}{\Delta\theta} - A^{up}\left(u^{n+1}_{(j,\frac{1}{2})}, \tilde{P}^{n+1}_{(j,N_\theta)}, \tilde{P}^{n+1}_{(j,1)}\right)\right).$$

We define the column vector $\mathcal{P}^n$ by $\mathcal{P}^n(k + (j-1)N_\theta) = \tilde{P}^n_{(j,k)}$ with $(j,k) \in \{1, ..., N_r\} \times \{1, ..., N_\theta\}$:

$$\mathcal{P}^n = \left(\tilde{P}^n_{(1,1)} \cdots \tilde{P}^n_{(1,N_\theta)}\, \tilde{P}^n_{(2,1)} \cdots \tilde{P}^n_{(2,N_\theta)} \cdots \tilde{P}^n_{(N_r,N_\theta)}\right)^T,$$

and the diagonal matrices $U^\pm_{j+\frac{1}{2}} \in M_{N_\theta}(\mathbb{R})$ for $j \in \{1, ..., N_r\}$:

$$U^\pm_{j+\frac{1}{2}} = \begin{pmatrix} \ddots & & & & \\ & (u^n_{(j+\frac{1}{2},k-1)})^\pm & & & \\ & & (u^n_{(j+\frac{1}{2},k)})^\pm & & \\ & & & (u^n_{(j+\frac{1}{2},k+1)})^\pm & \\ & & & & \ddots \end{pmatrix}.$$

Moreover we set:

$$\mathcal{B}^n = \begin{pmatrix} U^+_{\frac{3}{2}} & U^-_{\frac{3}{2}} & & & \\ & \ddots & \ddots & & \\ & & U^+_{j+\frac{1}{2}} & U^-_{j+\frac{1}{2}} & \\ & & & \ddots & U^-_{N_r-\frac{1}{2}} \\ & & & & 0 \end{pmatrix} - \begin{pmatrix} 0 & & & & \\ U^+_{\frac{3}{2}} & \ddots & & & \\ & U^+_{j-\frac{1}{2}} & U^-_{j-\frac{1}{2}} & & \\ & & \ddots & \ddots & \\ & & & U^+_{N_r-\frac{1}{2}} & U^-_{N_r-\frac{1}{2}} \end{pmatrix}$$
$$+ \begin{pmatrix} \frac{1}{r_1^2}B^n & & & & \\ & \frac{1}{r_2^2}B^n & & & \\ & & \ddots & & \\ & & & \frac{1}{r_{N_r-1}^2}B^n & \\ & & & & \frac{1}{r_{N_r}^2}B^n \end{pmatrix}, \tag{20}$$

Furthermore the flux boundary condition on $C(0,R)$ gives the following right hand side column vector of length $N_r\, N_\theta$:

$$\mathcal{R}^n = -\begin{pmatrix} 0 \\ \vdots \\ 0 \\ (\frac{\mu_k^{n+1}-\mu_k^n}{\Delta t})_k \end{pmatrix}.$$

At each time step we have

$$\frac{\mathcal{P}^{n+1}-\mathcal{P}^n}{\Delta t} = -\frac{1}{\Delta r^2}\mathcal{A}\mathcal{P}^{n+1} - \frac{1}{\Delta r}\mathcal{B}^{n+1}\mathcal{P}^{n+1} + \frac{1}{\Delta r}\mathcal{R}^n.$$

We use a standard numerical method to invert the matrix $\mathcal{A} + \Delta r\, \mathcal{B}^{n+1} + \frac{\Delta r^2}{\Delta t}\, I_{N_r N_\theta}$ and then resolve at each time step

$$\mathcal{P}^{n+1} = \left(\mathcal{A} + \Delta r\, \mathcal{B}^{n+1} + \frac{\Delta r^2}{\Delta t}\, I_{N_r N_\theta}\right)^{-1} \left(\frac{\Delta r^2}{\Delta t}\mathcal{P}^n + \mathcal{R}^n\right).$$

1.12 Hilbert transform

In the half-plane case, the Hilbert transform $\mathcal{H}$ is defined by
$\mathcal{H}\mu(t,x) := \dfrac{1}{\pi}\text{p.v.} \int_{\mathbb{R}} \dfrac{1}{x-y}\mu(t,y)\, dy.$

Detailed Experimental procedures.

Yeast strains.

Standard yeast manipulation methods were used. Strains used in this study are listed in S1 Text, Table A. All the strains constructed in this study are derivatives from JYL03 and JYL04 (W303 background) unless otherwise specified. Strains with total deletions or partial C-terminal deletions of genes, or with C-terminal insertion of fluorescent proteins, were produced according to standard methods [8]. Cassettes were amplified by PCR from plasmids made from the pFA6a backbone (most fluorescent protein cassettes come from plasmids produced by K. Thorn [9]) with a pair of primers that included 50 to 70 bp upstream and downstream of the targeted genomic region and integrated into the genome by homologous recombination.

Microfluidic chambers.

The design and fabrication of the chambers was based on regular soft lithography techniques (reviewed in ref. [10]). Such chambers are now popular tools to study cell response to temporally and spatially well controlled biochemical stimulations [11, 12]. Chambers to produce exponential and linear serial dilutions using advection chaotic mixers were based on previously described designs [13, 14]. In brief, various designs were drawn using CleWin (WieWeb Software, The Netherlands; see S1 Text, Fig A for chamber designs) and printed on high resolution transparencies. They were then transferred onto photo-resist resins, spin-coated on silicate wafers with the desired thickness, by UV insolation through the transparency. The resulting 3D structures were then used to mold polydimethoxysiloxane (PDMS) chambers, reviewed in [15]. Inlets and outlets were dug into the PDMS to allow access to the micro-channels and the channels were closed by bonding the PDMS onto a coverslip using plasma activation (20 sec exposure to an air plasma, Harrick Plasma, Ithaca, NY). At this stage, the chambers can be stored for weeks.

The day before using a chamber, channels were coated with a triethoxy-siloxane-aldehyde (Bio-Conext, PSX 1055, from UCT) to allow covalent binding of Concanavalin A, a lectin which binds strongly to the yeast cell wall. Chambers were first filled with ethanol until no bubbles were left, then a solution of ethanol containing 1% BioConext was introduced and incubated at room temperature for 4 hours. The channels were then heavily washed with ethanol, water, and finally with phosphate-buffered saline (PBS). A solution of PBS containing 0.1 mg/ml concanavalin A was flowed into the channels and incubated overnight at 4°C. Chambers were rinsed with PBS before adding the cells.

Cell cultures were grown overnight to saturation and then allowed to resume exponential growth by diluting them 50-fold into fresh growth medium, and incubating them for 3 hours at 30°C before the experiment. They were then treated as follows : if a single strain was to be studied in a given chamber, cells were rinsed twice in PBS, sonicated for 1min, counted and diluted to about 2 × 107 cells/ml in PBS, and flowed into the chamber (a micro-pipette tip containing the cells was inserted in the outlet and aspiration was produced from the inlet). Cells were incubated for 30 minutes at room temperature in the chambers. The micropipette tip was then removed and unattached cells were gently aspirated from the outlet before fresh tubing was inserted into the inlets and outlets. When several strains were to be studied in parallel in a given chamber, they were differentially stained prior to binding to the chamber. Cells were rinsed with water and incubated for 5 minutes in a solution of 0.1M sodium bicarbonate

containing an N-hydroxy succinimide (NHS) functionalized dye (usually A350 and Cascade blue, from Molecular Probes, which can be imaged with DAPI and CFP filers, respectively, and are thus compatible with imaging of YFP for promoter activity measurement or protein localization, and Texas-Red for measure of pheromone concentration - see below for more details). The reaction was stopped adding YPD (1% Yeast extract, 2% peptone, 2% dextrose) and cells were then rinsed in PBS, sonicated and flowed into the chamber as described above.
Two solutions were prepared to flow into the chamber: one containing only complete synthetic growth medium and the other containing the same growth medium plus the desired concentration of α-factor and 200 μg/ml of Texas Red conjugated dextran (MW 3000, Molecular Probes). 0.1% BSA was added to both solutions to prevent binding of pheromone and dextran to the walls of the tubing and chamber (importantly, prior to use, the tubing was also incubated overnight at 4°C while filled with a solution of 1% BSA in water). 10ml of the solutions to be flowed through the chambers were placed in 50ml Falcon tubes, which have a large cross-section, so the height of the solutions and thus the flow rate (about 0.1ml/h) would not change significantly in the course of the experiment. For the exponential dilution chambers, the two solutions were added to bottles whose cross sections ensured that the height of both solutions would change similarly. We rejected the alternative of using syringe pumps on the basis of cost and potential pitfalls (like gradient instability due to stick-slip in the syringes). The delivery tubing was fixed to the tubes or bottles containing the solutions. Solution was sucked into the tubing, which were then connected to the inlets of the chamber. The tubing containing pheromone was inserted first and clamped, then the tubing containing only growth medium was inserted and allowed to flow one minute to make sure the channel initially contained only growth medium. This tubing was also clamped to avoid any strong flow during transport of the chamber to the microscope. Particular care was taken to avoid any bubbles in the entire flow system.
Once all the tubing was inserted, chambers were inverted so that the coverslip was uppermost and fixed on a glass slide using nail varnish. The slide was then mounted on a motorized upright microscope stage for recording and the solutions were placed on jacks with adjustable height, next to the microscope, to allow control of the flow rate. After the slide was mounted on the microscope, the growth medium was allowed to flow through the chamber for 1 h, to allow cells to resume growth and budding. In the meantime, the positions of several fields of cells along the chamber were saved using the Metamorph software that controlled the motorized microscope stage and recording was started. Once a first round of pictures at each position was acquired, the tubing containing pheromone was unclamped, allowing pheromone to enter the chambers. Using the fluorescent dextran in the pheromone flow, the flows were adjusted to be equal at the point where they first encountered each other in the chamber (S1 Text, Fig A and C). We recorded the fluorescent dextran profile (as well as cellular behavior), both to calculate the local pheromone concentration and to check the temporal and spatial stability of the α-factor concentration during the experiment (S1 Text, Fig C).

Microscopy.

Time-lapse movies were acquired using a Nikon upright microscope, a motorized XYZ stage (Prior), a piezoelectric device from Princeton Instruments placed between the objective turret and the objective, a CCD camera (CoolsnapHQ, Roper), a motorized excitation and emission filter wheels (Ludl). The whole setup was controlled by Metamorph (Molecular Devices) software.
A typical time-lapse recording would acquire data from 3 different positions per minute of time-lapse. We used up to 100 positions (30 minutes between images of the same sample) for some gradient detection experiments, which required images at many

different concentrations, but only a few time-points at any position to measure the orientation of cells in the gradient, and about 40 positions for mating movies, corresponding to a time-lapse of 10 to 15 minutes. At each position, one DIC picture was acquired, followed by a fast Z-series acquired using the streaming mode and the piezo-electric device to move the objective, to record the localization of a fluorescently labeled protein (e.g. Spa2-YFP) throughout the cell volume with minimum exposure time. In flow chambers, a 40× objective was used, to record larger fields and thus more cells. An additional image was acquired to record the distribution of the fluorescent dextran, as a proxy for the local pheromone concentration. At each time-point, the stage was first centered on a 2μm diameter fluorescent bead using a home-made macro. This corrects for potential drift along the X-, Y-, or Z-axes during long recordings.

Image processing and analysis.

Cell behavior was analyzed semi-automatically. For each field that was recorded, macros in Metamorph automatically opened a time-series that overlaid a maximum intensity projection of the Z series of pictures of Spa2-YFP on the DIC image, and another time-series for the corresponding fluorescent dextran images. Interactive scripts facilitated the analysis and recording of various aspects of the behavior of each cell present in the recorded field (completion of cytokinesis, appearance and orientation of the polar Spa2cap, fluorescent dextran intensity around the cell, gradient strength and direction, etc.). These features were automatically saved in a table with one line per cell and one column per feature. These tables were analyzed using Matlab (Mathworks), to produce the statistical data shown in the figures.

Images of the fluorescent dextran were used to estimate the α-factor concentration. For each movie, an image of an homogenously fluorescent slide (obtained from Chroma) was first taken using the same acquisition parameters as for the experiment. This image was later used to inhomogeneous illumination (shading correction). For serial dilution chambers, fluorescent intensities were simply normalized by the maximum intensity in the undiluted channel and the minimum intensity in the reference channel that was never mixed with pheromone. For gradients produced using diffusion in simple laminar flow chambers, an additional correction was required due to the slight difference in the diffusion coefficients of the fluorescent dextran and α-factor, using a simple formula derived from the classical formula for time-dependent concentration profile of particles diffusing in a solution that is initially devoid of such particles, see [16], eq. 2.13 page 24. Time zero corresponds to the place where the two solutions meet in the chamber (the cross of the Y when there are two inlets), then the profile at a given distance from this point (along the flow axis) corresponds to the profile obtained at the time it took for the fluid to get there. To a first approximation (when the chamber is large enough and flow rates are slow enough), time in the classical equation can be replaced by the mean flow speed multiplied by the distance to the cross. The fluorescence intensity gives the dextran concentration, which can be used to find the position and thus the concentration of the pheromone. This can be done in a single step from the fluorescence value using the following equation:

$$C_p = 2C_{max}\left(1 + \mathrm{erf}\left(\mathrm{erfinv}(2F_{\mathrm{dex,norm}} - 1)\sqrt{\frac{D_{\mathrm{dex}}}{D_{\mathrm{pher}}}}\right)\right),$$

where $F_{\mathrm{dex,norm}}$ is the normalized fluorescence of dextran at given point, erfinv is a tabulated function (inverse of the error function), D_{dex} is the diffusion coefficient of dextran (21.3×10^{7} cm^2 s^{-1}) and D_{pher} is the diffusion coefficient of α-factor (32×10^{-7} cm^2 s^{-1}, after ref. [17]). C_{max} is the maximal concentration of pheromone in the chamber and C_p is the concentration of pheromone at the point where $F_{\mathrm{dex,norm}}$ was measured.

As S1 Text, Fig C shows, this correction is particularly significant for cells placed at the bottom of the gradient profile, which in many of our experiments corresponded to the region where cells polarized accurately in response to gradients, because it allowed us to also record cells placed in a similar relative gradient slope but at higher concentrations and thus displaying little or no gradient sensation.

Because both the cells and the fluid flowing past them are incompressible we needed to ask how much flow around the cells influenced the gradient they experienced in the chamber. Image analysis did not reveal any alteration of the gradient around the cells, but theory predicts that if the flow were fast enough, the gradient should be perturbed as the flow lines would part around the cell, thus bringing some fluid that first encountered the cell's equator towards its upper and lower poles. This would reduce the gradient actually felt by the cells for rapid flow, but diffusion would restore the gradient if the flow is slow enough. As we could not solve the problem analytically, we ran a realistic simulation of a cell in a chamber using Femlab (Mathworks). The result is shown in S1 Text, Fig C. For the mean flow rate used in our experiments, there is a very slight alteration of the gradient profile around the cells, so that instead of the minimum and maximum α-factor concentrations being felt at points on the cell periphery that were located at $90°$ relative to the flow axis, they were obtained at points displaced from these positions by less than $30°$ for the highest flow rate estimated for our experiments. The distribution of angles of between the shmoo and gradient axes in the gradient sensation regime is also shown in S1 Text, Fig C. It reveals a shift of the mean axis of polarization by an angle of approximately 15 degrees to that of the expected gradient direction, which we corrected for in all the gradient sensation measures we report. Note also the asymmetry of the distribution, which is expected because the gradient is sharper on the side of the cell away from the flow and is shallower on the side facing the flow.

S1 Text, Table A: Strains used in this study.

Strain Name	Bkgd	MAT	Relevant Genotype
MP 0384	W303	**a**	bar1△::ADE2 SPA2-YFP::HIS3
MP 1333	W303	**a**	bar1△::ADE2 cln1△::hisG cln2△ cln3△::LEU2 TRP1::PMET3-CLN2 SPA2-YFP::HIS3

S1 Text, Figure Legends

S1 Text, Fig A: Microfluidic chamber design.

1) Chambers were drawn using CleWin software. The chamber on the left has four independent channels with two inlets for each channel. It allowed us to run experiments in parallel with different gradient profiles or different strains. It produces a simple, exponential diffusion gradient. The chamber in the middle contains a section with chaotic mixers (see Experimental Procedures) producing a linear dilution: two entry channels are split into three channels (1, 1/2, 0) which are then split into 4 channels (1, 2/3, 1/3, 0) etc. up to seven channels (which gives 1, 5/6, 2/3, 1/2, 1/3, 1/6, 0). The channels are then brought together in a larger one in which diffusion can occur, giving rise to an approximately linear gradient. The width of the channel diminishes to provide various slopes. The "chip" comprises two independent chambers to run two parallel experiments. The chamber on the right also contains chaotic mixers, but channels are just sequentially split in two, providing an exponential dilution with a coefficient given by the ratio of the width of the buffer channel and the crossing channel. The chamber shown is meant for a dilution by two (channels of equal width). It produces fractions of the input concentration from 1 to 1/1024 with 10 intermediary dilutions by two, thus spanning three orders of magnitude in a single flow chamber. The channels are then brought together to allow the formation of an approximately exponential gradient.
2) Images from a typical experiment using a linear dilution chamber are shown. A fluorescent dextran was included in the pheromone-containing medium and fluorescent images were acquired with a 10× objective. Sections I to VI refer to A) and indicate which part of the chamber was imaged. Numbers on section I are theoretical dilutions. The graphs on the right show the measured fluorescence intensities as a percentage of the input fluorescence. Between section I and II, the gradient is not completely smoothed by diffusion. Sections III and IV display smooth, approximately linear gradients with two different slopes, reflecting the width of the channel at these points. Section V and VI are equivalent to III and IV, but with an approximately exponential gradient.

S1 Text, Fig B: Morphological and transcriptional responses to α-factor in bar1Δ cells.

1) Cells in micro-channels at various pheromone concentrations. The images overlay differential interference contrast and Spa2-YFP images. Without pheromone (left panel), all cells have budded (B) after a few hours (times are shown at the lower right corner in hr:min). Around 1nM, most cells are unpolarized and unbudded after 4h of exposure to pheromone, whereas they have all grown into shmoos (S) after 3h in 6nM (right panel). The intensity of the red dye indicates the concentration of the pheromone.
2) Details of cell behavior at various spatially uniform pheromone concentrations. All cells express Spa2-YFP and fluorescence and DIC images were merged. Note that the interval between images is different at different α-factor concentrations. To aid comparison between different treatments, time is indicated both as a color coded bar at the lower right corner and in hr:min at the lower left corner of each picture. White arrow heads show the first fully focused polar cap. At 6 and 20 nM, arrows also point to successive polar caps. For each cell, time 0 is set at the end of the first cytokinesis (when Spa2 leaves the bud neck) after the onset of pheromone treatment. At pheromone concentrations below 0.9 nM, cells arrest transiently and then bud, at 0.9 nM, one cell buds and the other cell shmoos, and above 0.9 nM, all the cells shmoo. Scale bars in A and B are 10 μm.

S1 Text, Fig C: Measuring concentrations in flow chambers.

1) Stability of pheromone gradients. Cells are very sensitive to variations in pheromone concentration. We thus recorded images of the fluorescent dextran throughout experiments and only used data from experiments displaying less than 10% variation in concentration during the experiment. The graph shows an example of a recording in a linear gradient chamber, over 10 hours. One image was acquired every 20 min. All measures are overlaid, so the line thickness gives the maximum deviation at each position. The insert shows the concentration over time at a position 60 μm from the lower side of the gradient, corresponding to about 1nM pheromone.
2) Calculation of pheromone concentrations. As explained in Experimental Procedures, the difference between the diffusion coefficients of the fluorescent dextran used for measurements and α-factor requires a correction in certain parts of the gradient. The graph on the left shows the concentration for both coefficients, calculated with a simple diffusion equation assuming a homogeneous flow speed, for a 2 inlet chamber, at a distance of 1cm from the point where the two flows join, with a flow speed of 2.5 mm/s. The graph on the right shows an example of gradient measured from a chamber and a fit with the equation used for dextran diffusion (the fitting parameter is the time from the point where the two flows first meet (the cross), which is equal to the distance divided by the flow speed). The red line is the corresponding pheromone concentration calculated using the same distance and flow speed and the pheromone diffusion coefficient. Note the agreement between the calculated and measured dextran concentration.
3) Simulations to test for effects of fluid flow on pheromone gradients. The top left drawing gives the parameters used in the simulation. The top right part is an image of the chamber with the color code corresponding to concentration. The lower part shows the concentration at the surface of a cell in the chamber. On the left, the concentration on the surface of an imaginary sphere through which the flow passes, with pheromone concentrations shown as heat map from highest (red) to lowest (blue). In the middle, the concentration on a hard sphere, representing a cell, for the flow rate used in our experiments. On the right, a hard sphere but with a 100$\times$ faster flow. In this case, the gradient on the cell surface is strongly affected by the presence of the cell and does not correspond to the gradient in the bulk fluid flow.
4) Distribution of shmoo angles relative to predicted gradient. The graph shows the distribution of angles for shmoos in a gradient. The center of the distribution is slightly off-center and follows the concentration profile of α-factor around the periphery of the cell (in green) obtained by simulation.

S1 Text, Fig D: Morphology and timing of response to pheromone gradients.

1) A field of cells which have been exposed to a gradient of pheromone (red). Concentrations along the gradient are indicated and a schematic representation of stereotypical cell behavior at various concentrations is shown below the micrograph. Cell polarity was recorded by the distribution of Spa2-YFP (shown in green). See Supplementary Movie.
2) Cumulative distributions of the angles between buds (left) or shmoos (right) and pheromone gradients, for various ranges of the mean pheromone concentration experienced by cells. Note that at concentrations below 0.45 nM or above 4 nM, 30% or

more of the cells polarize away from rather than towards the pheromone gradient.

S1 Text, Fig E: Gradient steepness has little effect on pheromone response.

1) The accuracy of gradient detection as a function of the relative slope of the gradient, for low ($<$ 2 nM, blue) and high ($>$5 nM, red) mean α-factor concentrations. The slope is expressed as the concentration difference between the two sides of the cell divided by the mean pheromone concentration the cell experienced.
2) Even in very steep gradients, *bar1*Δ cells cannot respond to gradients whose mean α-factor concentration exceeds 5 nM. A pair of *bar1*Δ cells responding to the very steep pheromone gradients that are created where pheromone-containing medium first encounters pheromone-free medium. The upper image shows the chamber in which medium flows from left to right and the lower images are higher magnification views of the boxed cell over time (hr:min). The cell body was initially located at pheromone concentrations that allowed gradient detection (mean pheromone concentration $\leq$ 3 nM), but because the gradient was very sharp, as the shmoo grew towards higher concentration, the mean concentration over the cell body increased, the shmoo stopped growing and the cells finally formed a second shmoo (arrowheads at 09:00 and 11:00), which grew up the gradient. Because these sharp gradients are difficult to measure (out of focus light from the high concentration region bleeds into the low concentration regions), we calculated the pheromone concentration gradient from the flow speed, diffusion concentration of the pheromone, and position of the cells as shown in the graph on the right.

S1 Text, Fig F: Sensitivity analysis of the model.

1) For different correlation lengths, namely for a number of sectors N varying from 5 to 120, we computed the dependence of the polarization delay on the mean concentration of pheromone. We observe that timing of polarization is not sensitive to the number of sectors.
2) For different values of the parameter λ (which represents the damping coefficient of the stochastic process describing κ), we computed the dependence of the polarization delay on the mean concentration of pheromone. We observe that polarization delay is not sensitive to the parameter λ.
3) When we consider no cell-to-cell variability (i.e. $\delta = 0$), we can fit the biological polarization delay but with a zero standard deviation. Such a result is not in good agreement with the experimental data.
4) When we omit cell-to-cell variability in the model (i.e. $\delta = 0$), the biological data of the fraction of cells which polarize are fitted with less accuracy than when we put some cell-to-cell variability.

S1 Text, Fig G

1) For different levels of Gaussian noise in the initial conditions for Cdc42 concentration, we compute the evolution of the timing of polarization with respect to the mean concentration of pheromone. We observe that polarization time doesn't depend strongly on the choice of the initial condition.
2) For different levels of Gaussian noise in the initial conditions, centered on the cell membrane, we compute the gradient detection. We observe that gradient detection depends strongly on the choice of the initial condition.
3) Timing of shmooing in Pheromone gradients. We observe that cells shmoo faster when they are submitted to a gradient than in uniform pheromone concentration.
4) Size of a shmoo. We observe that the length of a shmoo is about the third of the

total length of the cell.

S1 Text, Fig H

1) We observe that for $\lambda^{-1} = 10^2$ h the detection of the gradient with our model is much better than the one observed in the data. On the contrary, for $\lambda^{-1} = 10^{-6}$ h the detection of the gradient with our model is much worse than the one observed in the data.
2) Cells only shmoo at their prospective bud sites at unphysiologically high concentrations of α factor. The site of prospective budding can be identified by the location of Spa2 immediately after cell division, and we measured the angle between this spot and the site of shmoo emergence. The data show a representative experiment and each point averages $\cos\theta$ for at least 40 cells. The solid red curve for the mean value of $\cos\theta$ is hand-drawn. Without α-factor, the average of $\cos\theta$ is 0.85 (the broken red line). At each pheromone concentration, the standard deviation of $\cos\theta$ is shown as a black dot, and the value in the absence of pheromone is shown as a dashed black line. The grey stripe shows the range of sample values (mean $\pm$ 3 standard deviations) for the standard deviation of $\cos\theta$ for samples of 40 randomly drawn angles, showing that experimental distribution of polarization angles is nearly random over a wide range of pheromone concentrations. The highest pheromone concentration where 100% of the cells bud and the lowest concentration where 100% of the cells shmoo are indicated.

References

1. Evans LC. An Introduction to Stochastic Differential Equations. AMS; 2013.

2. Alberts B, Johnson A, Lewis J, Raff M, Roberts K, Walter P. Molecular Biology of the Cell, Fourth Edition. 4th ed. Garland Science; 2002. Available from: http://www.amazon.com/exec/obidos/redirect?tag=citeulike07-20&path=ASIN/0815332181.

3. Altschuler SJ, Angenent SB, Wang Y, Wu LF. On the spontaneous emergence of cell polarity. Nature. 2008 Aug 14;454:886–890.

4. Csikász-Nagy A, Gyorffy B, Alt W, Tyson JJ, Novák B. Spatial controls for growth zone formation during the fission yeast cell cycle. Yeast. 2008;25(1):59–69. Available from: http://dx.doi.org/10.1002/yea.1571.

5. Jenness DD, Burkholder AC, Hartwell LH. Binding of alpha factor pheromone to Saccharomyces cerevisiae **a** cells: dissociation constant and number of binding sites. Mol Cell Bio. 1986;6(1):318–20.

6. Marco E, Wedlich-Soldner R, Li R, Altschuler SJ, Wu LF. Principles for the dynamic maintenance of cortical polarity. Cell. 2007;129(2):411–420.

7. Kukulski W, Schorb M, Kaksonen M, Briggs JAG. Plasma Membrane Reshaping during Endocytosis Is Revealed by Time-Resolved Electron Tomography. Cell. 2012;150(3):508 – 520. Available from: http://www.sciencedirect.com/science/article/pii/S0092867412007842.

8. Longtine MS, Mckenzie A, Demarini DJ, Shah NG, Wach A, Brachat A, et al. Additional modules for versatile and economical PCR-based gene deletion and modification in Saccharomyces cerevisiae. Yeast. 1998;14(10):953–961.

9. Sheff MA, Thorn KS. Optimized cassettes for fluorescent protein tagging in Saccharomyces cerevisiae. Yeast. 2004;21(8):661–670. Available from: http://dx.doi.org/10.1002/yea.1130.

10. Whitesides GM, Ostuni E, Takayama S, Jiang X, Ingber DE. SOFT LITHOGRAPHY IN BIOLOGY AND BIOCHEMISTRY. Annual Review of Biomedical Engineering. 2001;3(1):335–373. Available from: http://dx.doi.org/10.1146/annurev.bioeng.3.1.335.

11. Hersen P, Mcclean MN, Mahadevan L, Ramanathan S. Signal processing by the HOG MAP kinase pathway. Proceedings of the National Academy of Sciences of the United States of America. 2008 May;105(20):7165–7170. Available from: http://dx.doi.org/10.1073/pnas.0710770105.

12. Taylor RJ, Falconnet D, Niemistö A, Ramsey SA, Prinz S, Shmulevich I, et al. Dynamic analysis of MAPK signaling using a high-throughput microfluidic single-cell imaging platform. Proceedings of the National Academy of Sciences of the United States of America. 2009 Mar;106(10):3758–3763. Available from: http://dx.doi.org/10.1073/pnas.0813416106.

13. Jiang X, Ng JMK, Stroock AD, Dertinger SKW, Whitesides GM. A Miniaturized, Parallel, Serially Diluted Immunoassay for Analyzing Multiple Antigens. Journal of the American Chemical Society. 2003 May;125(18):5294–5295. Available from: http://dx.doi.org/10.1021/ja034566+.

14. Stroock AD, Dertinger SKW, Ajdari A, Mezic I, Stone HA, Whitesides GM. Chaotic Mixer for Microchannels. Science. 2002 Jan;295(5555):647–651. Available from: http://dx.doi.org/10.1126/science.1066238.

15. McDonald JC, Duffy DC, Anderson JR, Chiu DT, Wu H, Schueller OJA, et al. Fabrication of microfluidic systems in poly (dimethylsiloxane). Electrophoresis. 1999;(1):27–40.

16. Berg HC. Random Walks in Biology. Revised ed. Princeton University Press; 1993. Available from: http://www.amazon.com/exec/obidos/redirect?tag=citeulike07-20&path=ASIN/0691000646.

17. Segall JE. Polarization of yeast cells in spatial gradients of alpha mating factor. Proceedings of the National Academy of Sciences. 1993;90(18):8332–8336. Available from: http://www.pnas.org/content/90/18/8332.abstract.

S1 Text, Fig A

Chamber design

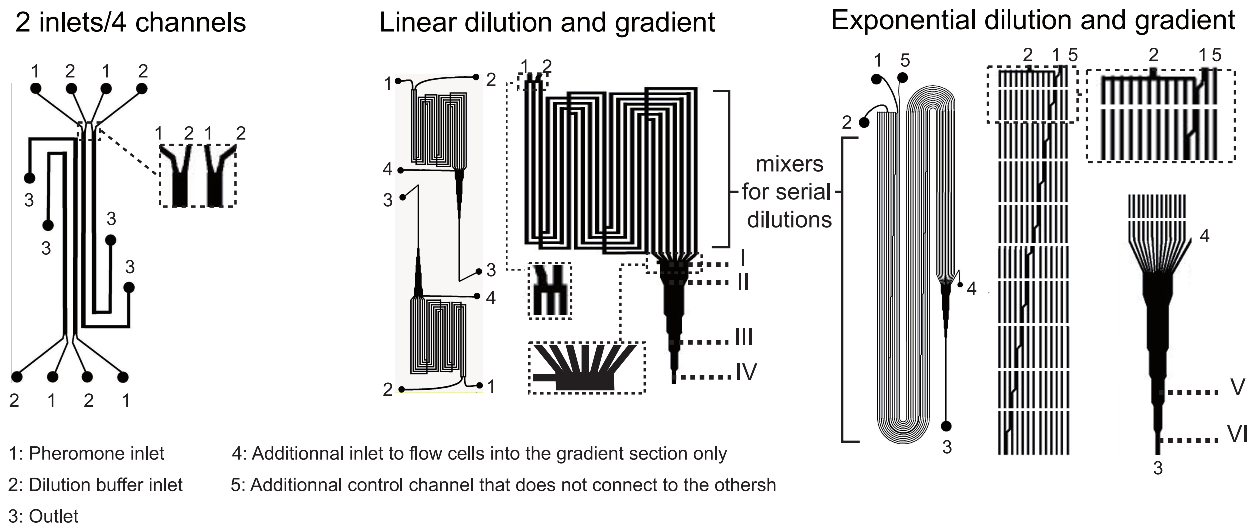

Dilution and gradient formation in a linear dilution chamber

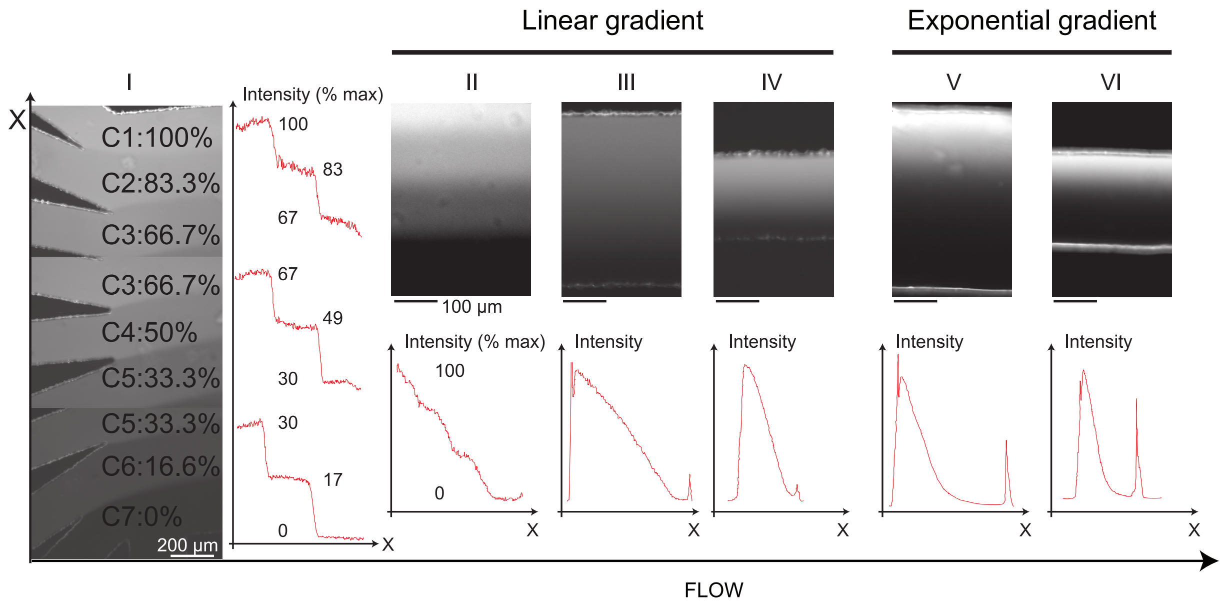

S1 Text, Fig B

Response to homogeneous fields

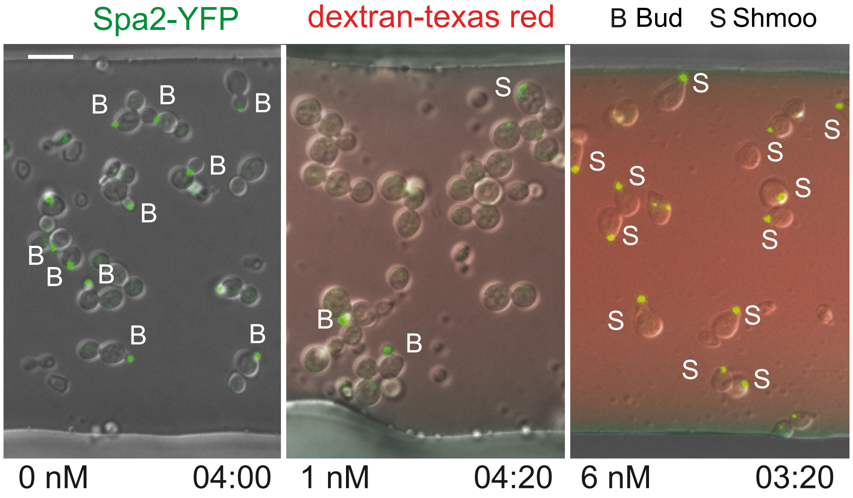

Timing of cellular responses in homogeneous fields

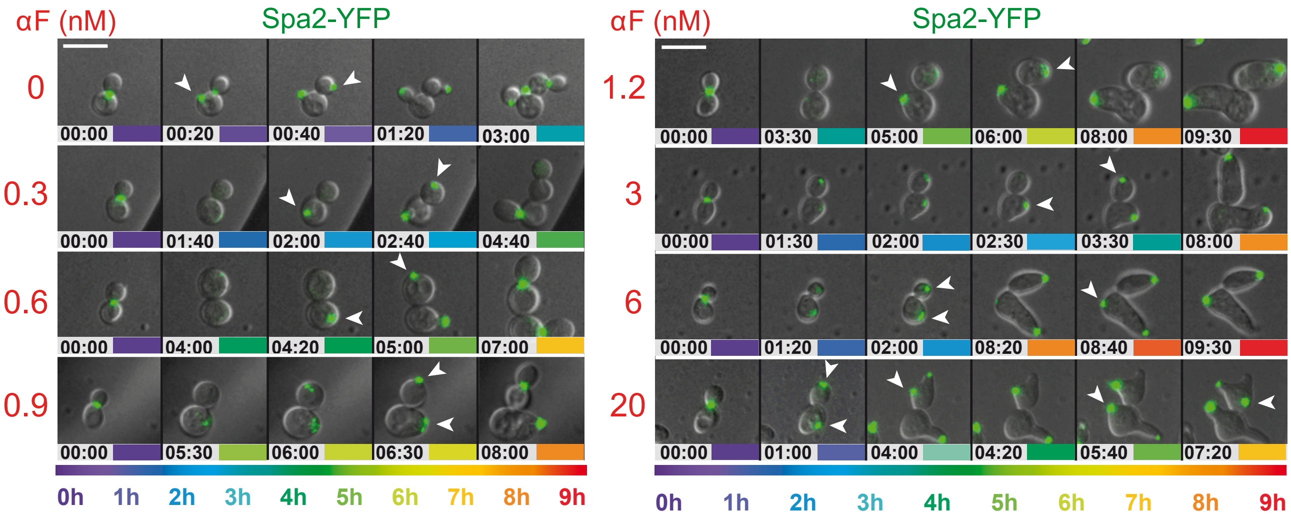

S1 Text, Fig C

Gradient stability

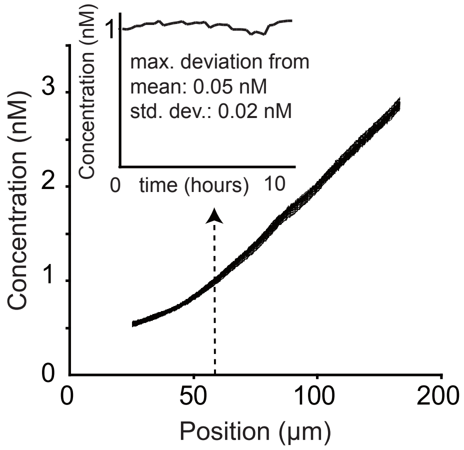

Correction for diffusion coefficient

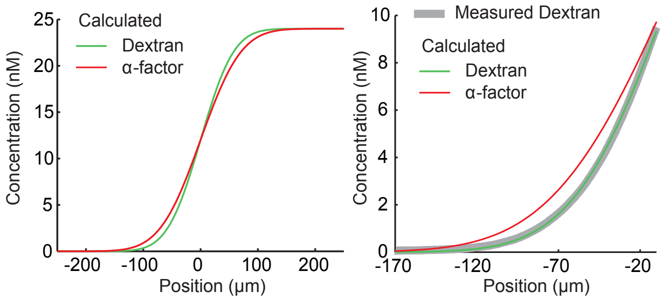

Effect of a cell on the gradient

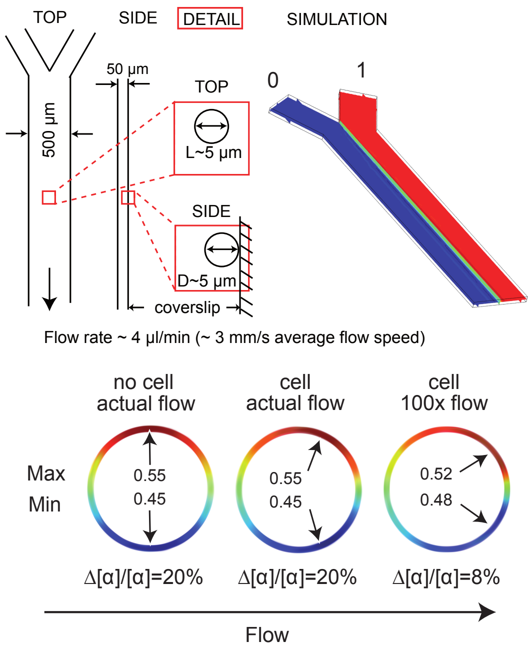

Distribution of shmoo angles

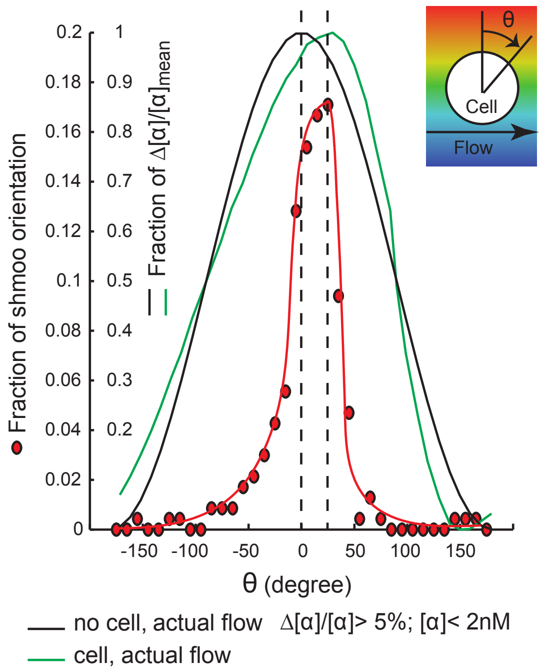

S1 Text, Fig D

Response to gradient fields

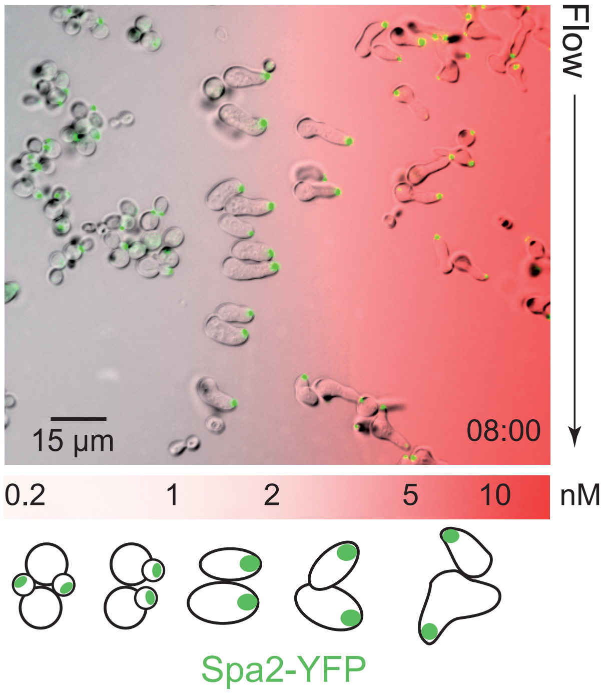

Directional response to gradient fields: cumulative fraction of cells

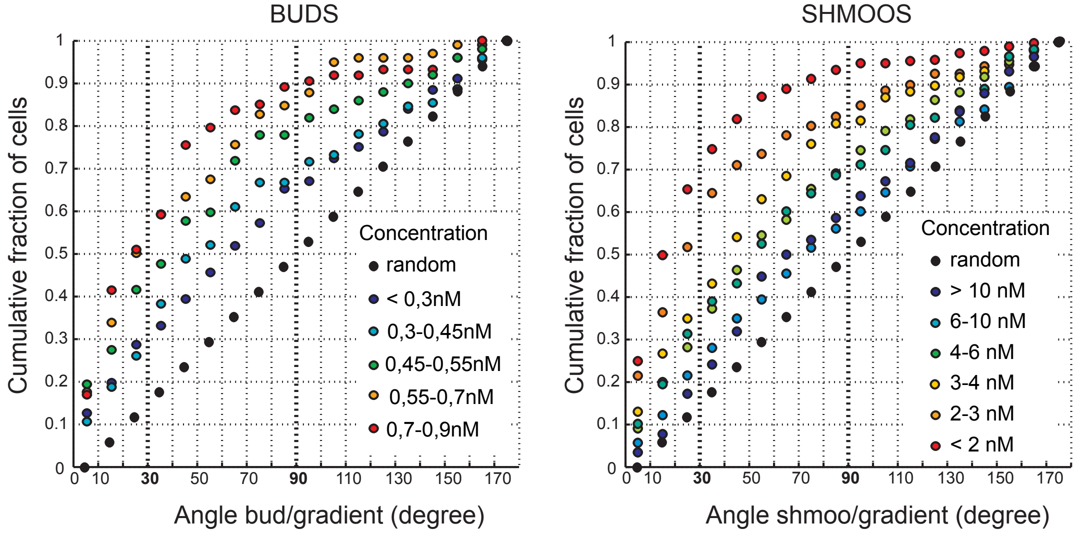

S1 Text, Fig E

Effect of gradient on directional response

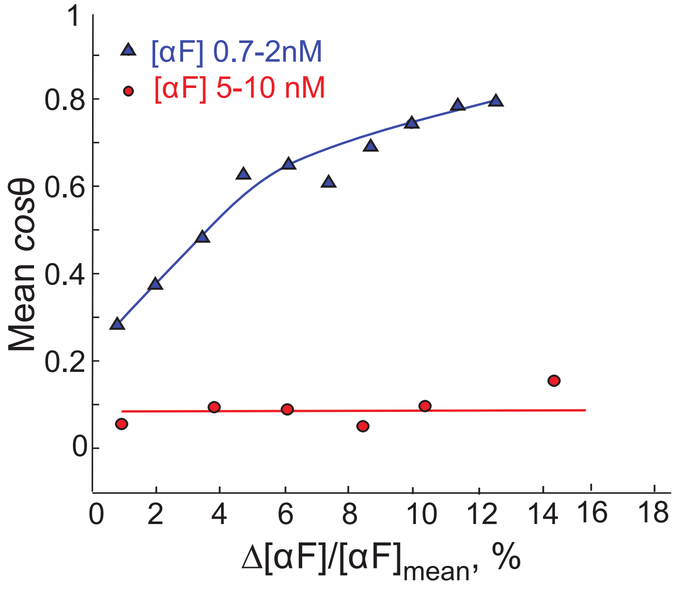

Bar1Δ cells in a sharp gradient

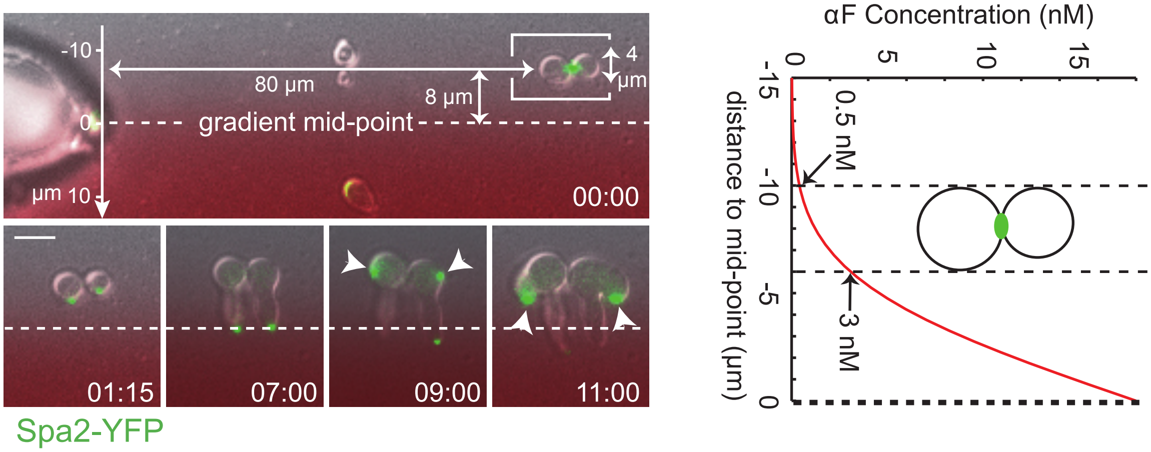

S1 Text, Fig F

Sensitivity to the number of sectors

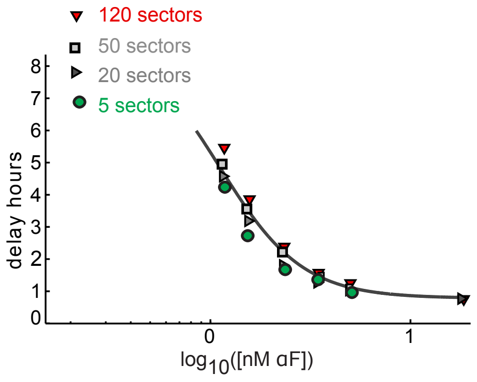

Sensitivity to the parameter λ

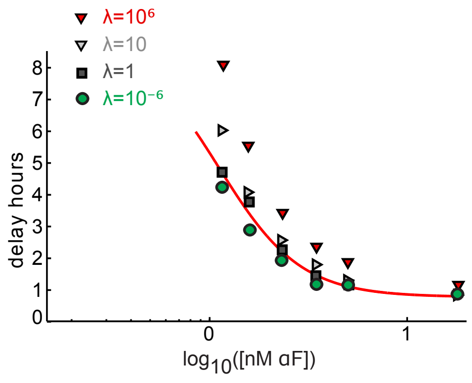

Timing of polarization without cell variability

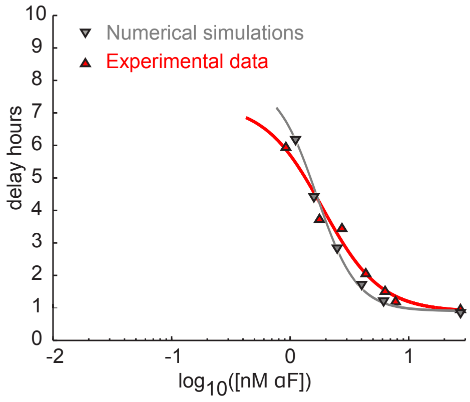

Fraction of cells which polarize without cell variability

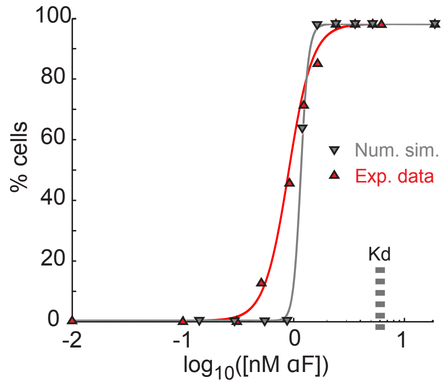

S1 Text, Fig G

Sensitivity to the initial condition in uniform concentration

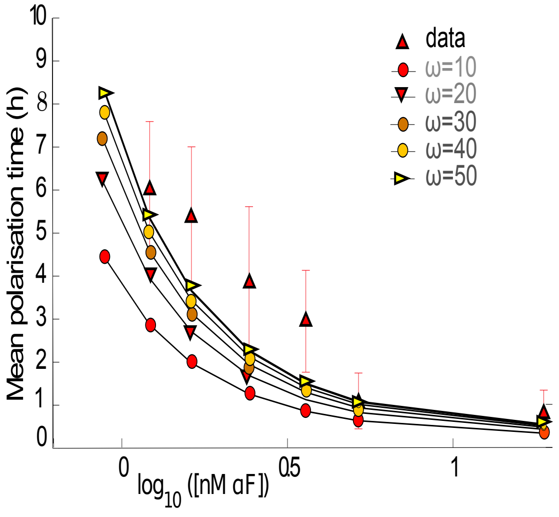

Sensitivity to the initial condition in gradients

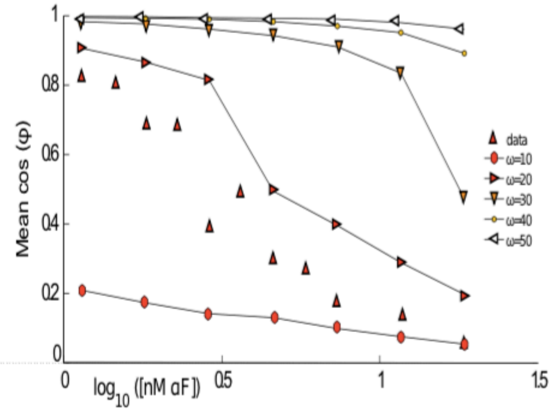

Timing of shmooing in gradient

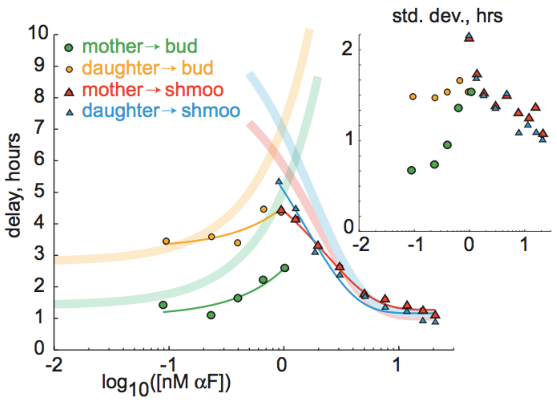

Size of a shmoo

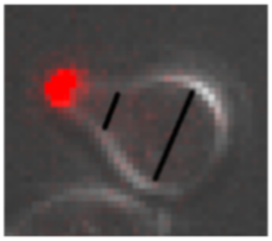

S1 Text, Fig H

Directional response

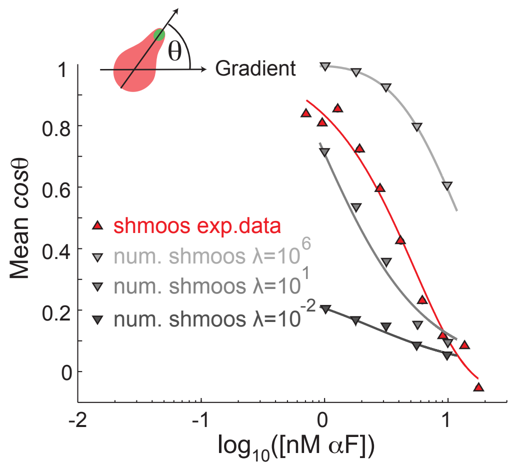

Gradient response

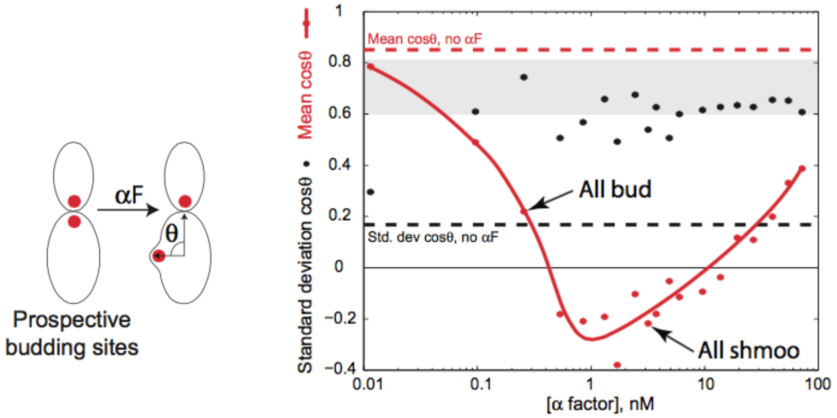

Supplement: S1 Text — (PDF) [file pcbi.1004795.s001.pdf]
